# Health system and environmental factors affecting global progress towards achieving End TB targets between 2015 and 2020

**DOI:** 10.7189/jogh.15.04004

**Published:** 2025-01-10

**Authors:** Haileab Fekadu Wolde, Archie CA Clements, Kefyalew Addis Alene

**Affiliations:** 1 School of Population Health, Faculty of Health Sciences, Curtin University, Bentley, Western Australia, Australia; 2 Geospatial and Tuberculosis Team, Telethon Kids Institute, Nedlands, Western Australia, Australia; 3 Institute of Public Health, College of Medicine and Health Sciences, University of Gondar, Gondar, Ethiopia; 4 School of Biological Sciences, Queen’s University, Belfast, UK

## Abstract

**Background:**

Health system and environmental factors play a significant role in achieving the World Health Organization (WHO) End Tuberculosis (TB) targets. However, quantitative measures are scarce or non-existent at a global level. We aimed to measure the progress made towards meeting the global End TB milestones from 2015 to 2020 and identify health system and environmental factors contributing to the success.

**Methods:**

We obtained data from ten different online data repositories and used principal component analysis to create domain-specific health system performance measures. We used radar charts and dumbbell plots to show the country's progress in ending TB with their health systems. Lastly, we used a linear regression model to identify key health systems and environmental predictors of the percent reduction in TB incidence and mortality.

**Results:**

There was a high variation in TB incidence and mortality reduction between countries and WHO regions. Of all countries included, 75 (39.3%) achieved more than a 20% reduction in TB incidence between 2015 and 2020. However, only 31 (16.2%) reached a 35% reduction in TB mortality. The European Region achieved the highest incidence reduction, exceeding the 2020 milestone with a 25% reduction. The African Region also made notable progress, achieving an 18% mortality reduction despite its relatively poor health systems. Health system factors, such as TB financing, TB-specific health service delivery, access to medicine, and governance, were significantly associated with TB mortality reduction between 2015 and 2020. Environmental factors, such as average annual temperature and air particulate matter concentration, were found to have a significant negative effect on TB incidence and mortality reduction.

**Conclusions:**

Weak health systems were identified as major barriers to achieving the End TB milestones in most high-burden countries. Hence, strengthening health systems with a special focus on TB financing, service delivery, and access to medicine in these countries should be prioritised to achieve global TB mortality reduction targets. Countries should follow WHO’s air quality guidelines and rapidly reduce carbon dioxide and other greenhouse gas emissions to mitigate the impact of environmental factors.

Tuberculosis (TB) is a substantial cause of mortality and morbidity and is the leading cause of death from a single infectious agent worldwide [1]. According to a recent World Health Organization (WHO) estimate, TB affects more than 10 million and kills nearly 1.5 million people globally every year [1,2]. It also causes 1.9% of total disability-adjusted life years lost worldwide [3]. Incidence of TB varies substantially across different WHO regions, for instance, the African region has the highest incidence with 220 cases per 100 000, while the European region is at the bottom with 25 cases per 100 000 population [4]. Approximately 87% of the world’s TB cases occur in the 30 high TB-burden countries [5], and according to a 2020 WHO report, China, India, and Indonesia accounted for 42.9% of the global caseload [6].

The United Nations (UN) Sustainable Development Goals and the WHO end TB strategy share a common goal to end the global TB epidemic, with targets to reduce TB incidence by 80% and TB deaths by 90% by 2030, relative to baseline levels in 2015 [7,8]. The WHO End TB strategy has two milestones to be achieved by 2020 and 2025, with the first aimed at reducing TB incidence by 35% and TB death by 20% between 2015 and 2020 [8]. However, the WHO progress report indicated that the world is far from reaching these targets, as TB incidence decreased by only 11% and TB deaths by 9.2% [4]. Among WHO regions, only the European Region exceeded the milestone for incidence reduction, while the African Region was close to the target with a 19% reduction. However, other regions showed small or no change in incidence between 2015 and 2020. Similar variations in the achievement of TB mortality reduction targets were also reported at regional and country levels [4]. Therefore, there is a need to investigate and understand the reasons for these variations.

TB control strategies have traditionally focussed on biological factors, although health system and physical environmental factors may also play a key role in achieving the End TB strategy milestones [9]. The WHO framework describes health systems in terms of six building blocks, which include service delivery, health workforce, health information systems, access to essential medicines, financing, and leadership or governance [10]. Well-functioning health systems are key to reaching global and national TB control targets [11], whereas weak ones hinder global efforts for TB control in developing countries [12]. Hence, achieving the targets of the end TB strategy requires a paradigm shift from focussed actions that gradually reduce the incidence of TB to enhanced, multisectoral actions that have been shown to drive down the epidemic at a rapid pace [8].

Climate change in recent decades has had a significant negative impact on the global environment, with the incidence of TB being significantly correlated with changes in regional environmental factors [13]. These factors include humidity [14,15], sunshine exposure [16], wind speed [15], air pollutants [14,15,17,18], temperature, and rainfall [14,15,19–21]. Incorporating physical environmental variables into models investigating ecological-level drivers of TB disease burden is essential, not only because they may confound the effects of health system factors, but also because their inclusion enables public health actions. These actions include forecasting the impacts of environmental changes, assessing vulnerabilities, evaluating available interventions, and formulating or adapting climate change and health adaptation plans [22] to mitigate their effects.

Although several regional studies have explored the correlation of health systems and environmental factors with TB control, there has been no such study on a global scale. Having evidence about the effect of these factors on the global and country-level progress towards achieving the global end TB targets is crucial in designing and operationalising an effective country-specific response to end the TB epidemic. Here we aimed to quantify the association between the health system and physical environmental factors on TB burden to identify the most important drivers and identify appropriate interventions to enable the achievement of the End TB strategic goals.

## METHODS

### Data sources and variables

We developed an ecological study design, where correlations between the incidence and mortality reduction and exposure variables were measured at a country level. The primary outcome and data on the exposure variables were assembled from multiple sources (Table S1 in the **Online Supplementary Document**). Our primary outcome measures were TB mortality and incidence reductions between 2015 and 2020. We calculated the percentage reduction in TB incidence and mortality by subtracting the 2020 value from the 2015 baseline value and dividing the difference by the baseline value. We obtained these data from the WHO global TB databases [23]. Additionally, we obtained health system variables from the World Bank Health and Nutrition Statistics [24], Demographic and Health Surveys [25], and the WHO Global Health Observatory [26], while we extracted the environmental variables, including climatic factors, from the World Bank Climate Change Knowledge Portal [27]. We selected potential health system and environmental factors based on the availability of the data and their relevance for TB control programmes.

We extracted country-level variables for a total of 194 WHO member states for the years 2015 and 2020. Of these countries, we excluded San Marino, Monaco, and North Korea due to a high rate of missing data, including the outcome variables, which may have caused significant uncertainty and bias. We have selected the 2015 and 2020 time points intentionally to align our study with the first WHO end TB milestone targets. The first milestone of the End TB strategy in 2020 has a target of a 20% reduction in TB incidence and a 35% reduction in the number of TB deaths, compared to the year 2015 as the baseline. Hence, we used two outcome variables to assess the progress towards achieving these targets: comparative reductions in TB incidence and TB mortality between 2015 and 2020. We used the human development index (HDI) of countries, which is an indicator that includes income, education and life expectancy [28], general health expenditure of countries, and level of TB burden in 2015, as control variables. We classified countries as having high and low TB burdens based on the 2016 WHO global TB report [29].

We extracted a total of 50 health system variables that are indicators for each building block of the health system and three environmental indicator variables for 2015 and 2020 (Tables S2 and S3 in the **Online Supplementary Document**). We then estimated the average values at the two time points for each variable, excluding any variable with missing data for more than 50% of the countries.

### Data consolidation and generating health system scores

We grouped health system variables based on the building blocks of the WHO health system framework, which includes health system financing, health service delivery, access to medicines, health system workforce and capacity, governance, and information systems [10]. We also included TB-specific building blocks, such as TB financing, TB-specific health service delivery, and TB-specific health service capacity. Additionally, we consolidate the available data within each domain of health systems using principal component analysis, determining the number of dimensions to be retained based on Kaiser’s rule and the variance explained by each dimension [30]. We retained all principal components with eigenvalues greater than one and included additional components when the cumulative variance explained remained below 50% (Table S4 in the **Online Supplementary Document**). Furthermore, we generated a single index representing each domain of the health system framework from the selected principal components, weighted by the amount of variance explained, and categorised countries into deciles for each building block. Finally, we summed the decile rank of countries for each health system building block to generate the overall health system score, ranging from 9 (minimum possible score) to 90 (maximum possible score), reflecting countries with the weakest and the strongest health systems, respectively.

### Missing data management

Since we sourced our data from multiple reputable databases, the probability of data being missing was most likely influenced by observable factors, such as the country's data reporting capabilities and overall health system infrastructure, which are captured in other observed variables from these comprehensive data sets. Hence, we assumed that data were missing at random after considering these factors and used multiple imputations by chained equations [31] to generate five complete data sets for each variable with missing values. We used the predictive mean matching method, the gold standard approach for imputation, to impute missing numeric variables and logistic regression for binary variables [32]. Lastly, we combined estimates from each complete data set using Rubin’s rule [33].

### Statistical analysis

We conducted a preliminary analysis to see the individual effect of all included health system indicators and environmental variables on the percentage reduction in TB incidence and mortality. We used dumbbell plots to visualise the change in incidence and mortality concerning the rank of countries based on the total health system score. We also implemented radar charts to compare the worst and best-performing countries in incidence and mortality reduction in each burden category concerning their score for each building block of the health system, and we plotted kernel-weighted local polynomial smoothed relationships between percentage reduction in TB incidence and mortality and total health systems scores for each WHO region. After checking all model assumptions, two similar linear regression models were fitted for incidence and mortality reduction. We put the general equation for both models as follows:



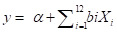



Where *Y* is the percentage reduction in TB incidence or TB mortality in 2020 compared to 2015, α is the intercept, *b* is a vector of covariate coefficients, and *X* is the matrix of 12 covariates which include nine health systems and three environmental variables.

We used backward stepwise regression to select variables and conducted sensitivity analysis by fitting separate regression models for high and low-burden countries. We adjusted models by including all countries for baseline TB burden, HDI, and current total health expenditure as a percentage of GDP, while adjusting models for specific burden categories only for HDI and current total health expenditure as a percentage of GDP. We fitted binary logistic regression models to assess the effect of each health system component and environmental factors on achieving the WHO End TB target for incidence and mortality, using the milestone one targets as cut-off points to generate binary outcomes. We considered health system and environmental factors with *P*-values less than 0.05 to have a significant association with the outcomes and used *β* coefficients with corresponding 95% confidence intervals (CIs) to report effect sizes. We assessed model performance using the coefficient of determination (*R*^2^).

## RESULTS

### Global progress

Of the 191 included countries, 75 (39.3%) achieved more than 20% reduction in TB incidence between 2015 and 2020 (**Table 1**). However, only 31 (16.2%) countries reached a 35% percent reduction in TB mortality. Additionally, 35 (68.6%) European countries achieved milestone one for TB incidence reduction, while 11 (23.4%) countries in the African Region reached a 35% reduction in TB-related deaths.

**Table 1:** Proportion of countries in each WHO region that achieved milestone one of the End TB targets

	>20% reduction in incidence		>35% reduction in mortality	
**WHO region (number of countries)**	**Yes, n (%)**	**No, n (%)**	**Yes, n (%)**	**No, n (%)**
All regions (191)	75	116	31	160
African Region (47)	14 (29.8)	32 (68.1)	11 (23.4)	36 (76.6)
Region of Americas (35)	9 (25.7)	26 (74.3)	0 (0.0)	35 (100.0)
Eastern Mediterranean Region (21)	6 (28.6)	15 (71.4)	2 (9.5)	19 (90.5)
European Region (51)	35 (68.6)	16 (31.4)	11 (21.6)	40 (78.4)
South-East Asian Region (10)	1 (10.0)	9 (90.0)	1 (10.0)	9 (90.0)
Western Pacific Region (27)	10 (37.0)	17 (63.0)	6 (22.2)	21 (77.8)

**Figure 1:**
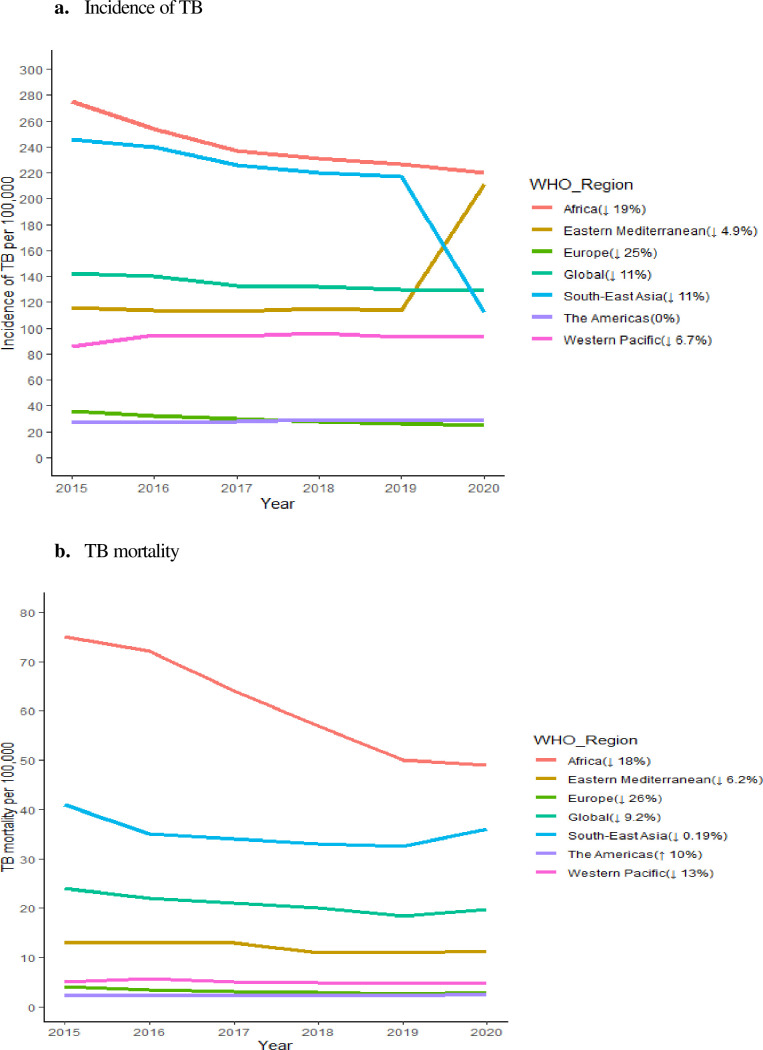
Trends of TB incidence and mortality change between 2015 and 2020 for each WHO region and globally. The percentage values in the bracket indicate the overall change in TB incidence and mortality. **Panel A.** TB incidence. **Panel B.** TB mortality. TB – tuberculosis.

Globally, TB incidence reduced by 11% between 2015 and 2020 (**Figure 1**, Panel A). The European Region was the only one to meet the 2020 end TB milestone, with a 25% reduction in incidence. Africa made the most progress among other regions, with a 19% reduction, while the Americas showed no progress. Global progress on mortality reduction is far behind the target, with only a 9.2% reduction (**Figure 1**, Panel B). The European Region had the largest reduction in mortality at 26%, followed by the African Region with an 18% decrease. In contrast, there was a 10% increase in mortality in the Americas Region. All the regions except for Africa showed an increase in mortality between 2019 and 2020.

### Health system strength

**Figure 2:**
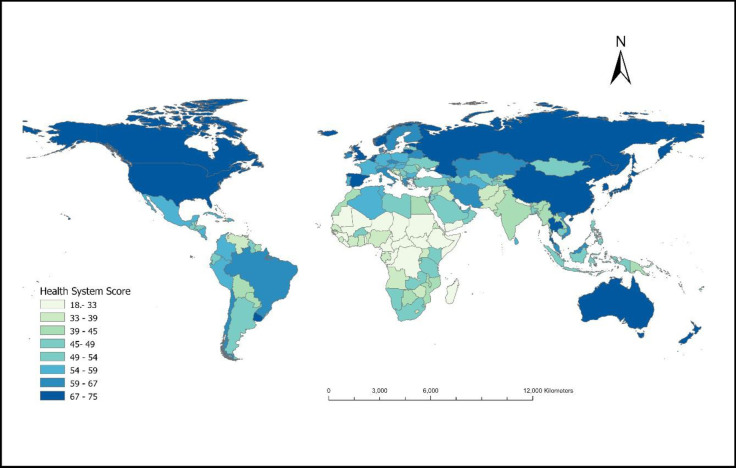
Global map showing the health system strength of countries based on their overall health system score.

Among all countries, Equatorial Guinea and the Central African Republic exhibited the weakest health systems, each with an overall health system score of 18, followed by South Sudan with a score of 20 (**Figure 2**; Table S5 in the **Online Supplementary Document**). In contrast, Estonia, the USA, and South Korea achieved the highest scores. Within the high TB burden countries, the Russian Federation and China had the strongest health systems, both scoring 73, followed by Thailand with a score of 72. The Central African Republic had the weakest health system in this category. The median health system scores were higher in countries with higher HDI (Figure S1 in the **Online Supplementary Document**).

### Regional progress

We observed an association between TB incidence reduction and the health system scores of countries, indicating that nations with higher reductions in TB incidence tend to have relatively better health systems. The WHO African Region has relatively poor health systems with an average health system score of 36.9 (95% CI = 34.0–39.8). Conversely, the European Region demonstrated the highest health system score among all regions, with an average score of 57.8 (95% CI = 55.1–60.5), and was the only region to achieve a 20% reduction in TB incidence between 2015 and 2020 (**Figure 1**). In both the Americas and European regions, there was a positive association between the percentage reduction in TB mortality and the health system scores of countries, as countries with better health systems generally reported higher reductions in mortality (Figure S2, Panels B and D in the **Online Supplementary Document**).

### Country-level progress: TB incidence

**Figure 3:**
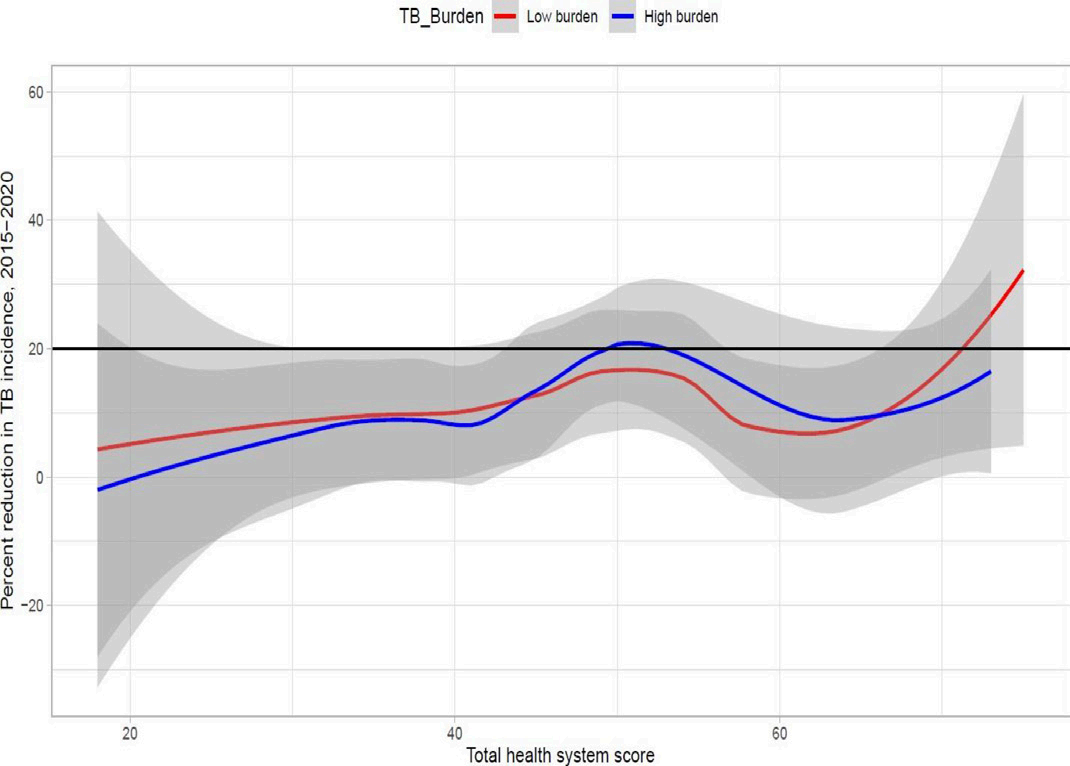
Kernel-weighted local polynomial smoothed relationship between percentage reduction in TB mortality and total health systems scores, categorised by the initial burden of TB. The horizontal black line represents the target of the WHO End TB strategy milestone (i.e. 20% reduction in TB incidence); the shaded area surrounding the regression lines represents the 95% CI for the change in TB incidence between 2015 and 2020. TB – tuberculosis.

Percent reduction in TB incidence generally showed a direct relationship with health system scores both in low and high-burden countries (**Figure 3**).

**Figure 4:**
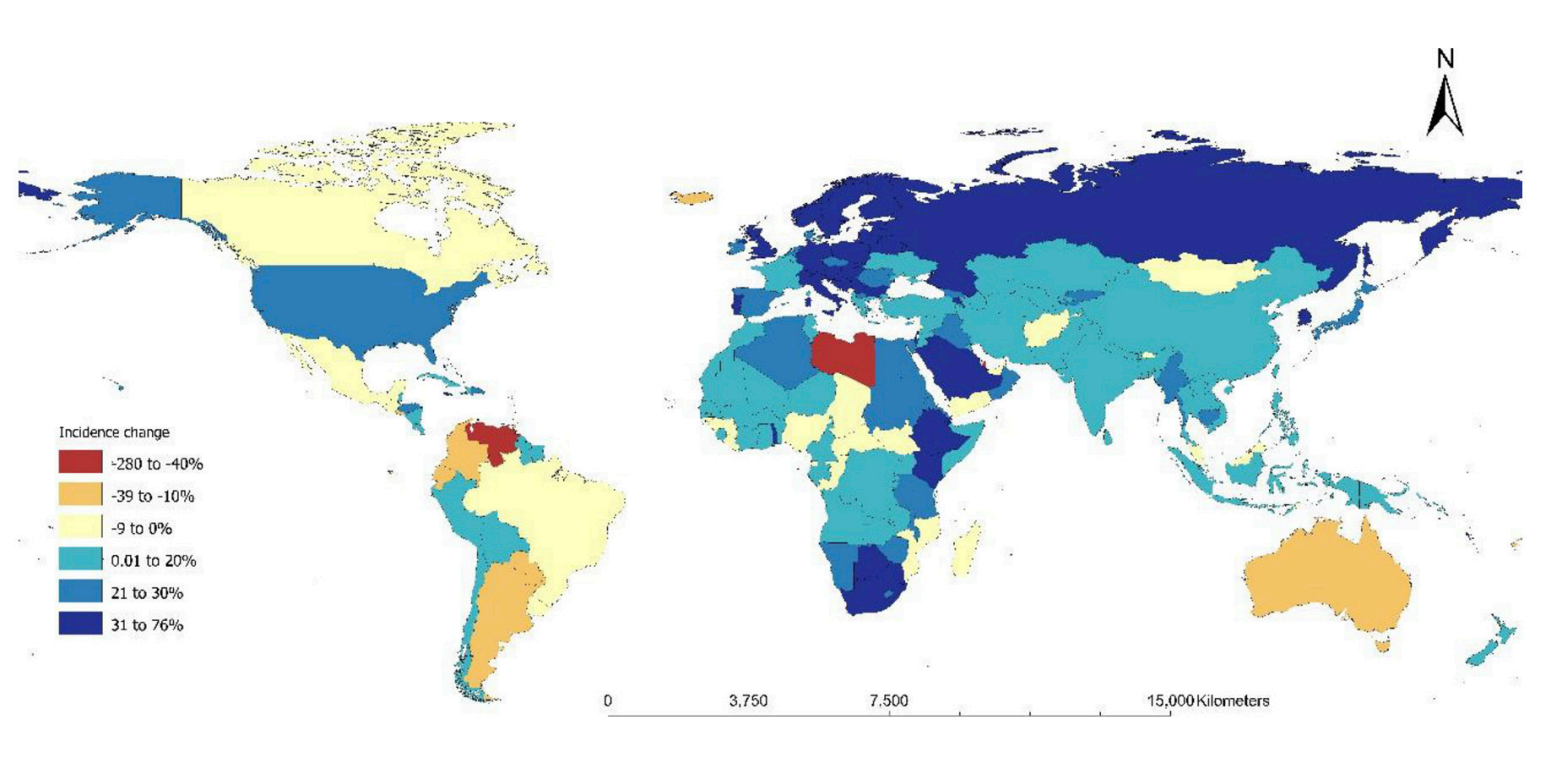
Global map showing the percentage change in TB incidence between 2015 and 2020 for each country. TB – tuberculosis

Between 2015 and 2020, TB incidence increased in 42 countries and showed no change in 7 countries, representing more than a quarter of the countries globally (**Figure 4**; Table S5 in the **Online Supplementary Document**). Over half of these countries (55%) had weak health systems, ranking in the lowest 50% of countries based on health system scores. However, there were notable exceptions to this trend. Australia and Canada, despite being ranked among the top ten countries for health system strength, experienced increases in TB incidence by 21.6% and 1.9%, respectively.

**Figure 5:**
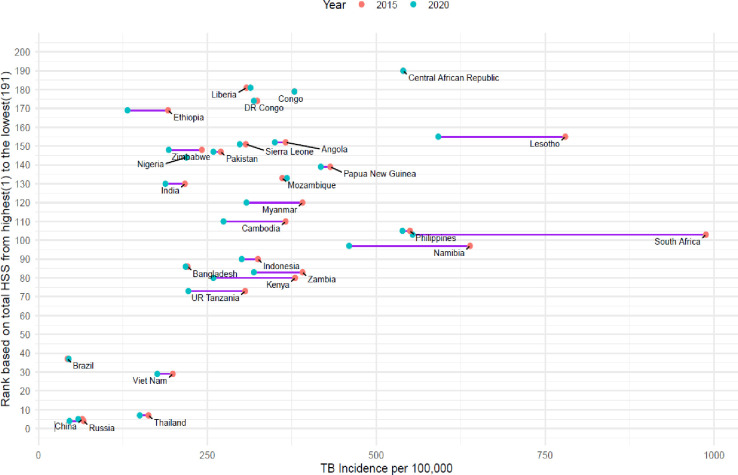
Dumbbell plot showing ranking based on total health system scores and change in TB incidence rate between 2015 and 2020 among high TB burden countries. The length of the purple line connecting the two points represents the extent of the change in TB incidence rates for each country between 2015 and 2020, expressed per 100 000 population. TB – tuberculosis.

Among high TB burden countries, only six (i.e. the Republic of Congo, Nigeria, Liberia, Central African Republic, Brazil, and Mozambique) showed no change or an increase in TB incidence (**Figure 5**). These countries were predominantly among the 30% of countries with the weakest health systems globally, except for Brazil, which is ranked 37th in terms of health system strength. In contrast, South Africa, Kenya, Ethiopia, and the Russian Federation demonstrated the greatest reductions in TB incidence within this category.

### Country-level progress: TB mortality

**Figure 6:**
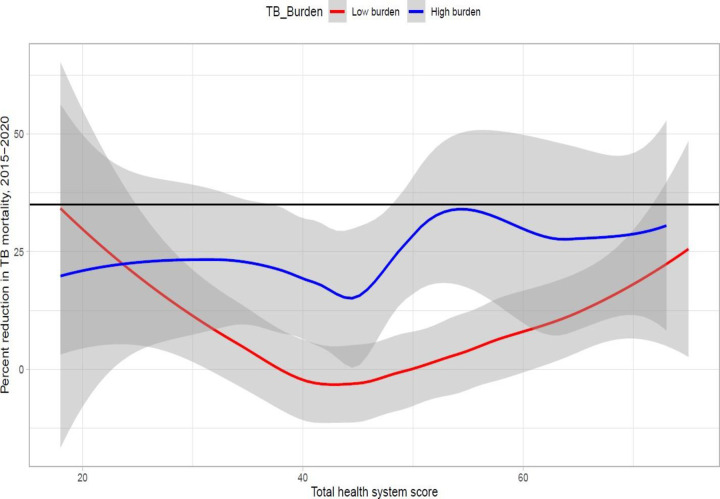
Kernel-weighted local polynomial smoothed relationship between percent reduction in TB mortality and total health systems scores, categorised by the initial burden of TB. The horizontal black line represents target of the WHO End TB strategy milestone (i.e. a 35% reduction in TB-related mortality between 2015 and 2020); the shaded area surrounding the regression lines represents the 95% CI for the change in TB mortality between 2015 and 2020. TB – tuberculosis.

The smoothed relationship between percentage reduction in TB mortality and health system score showed different relationships for high and low-burden countries (**Figure 6**). In low-burden countries, an inverse relationship was observed between the percentage reduction in TB mortality and health system scores for countries with scores ranging from 18 (the minimum score) to 40. However, this trend reversed for countries with health system scores above 40, where higher health system scores corresponded to increased percentage reductions in TB mortality.

**Figure 7:**
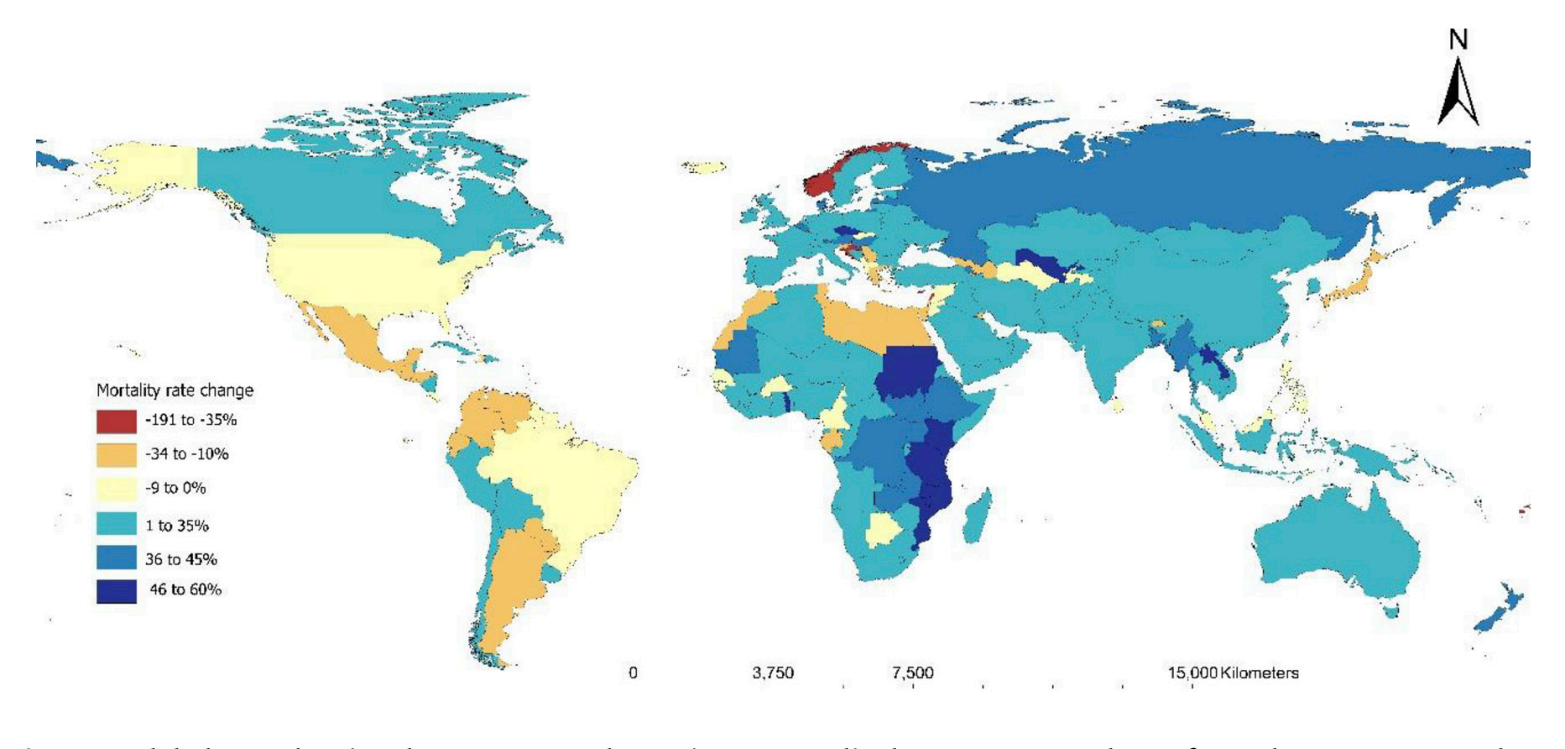
Global map showing the percentage change in TB mortality between 2015 and 2020 for each country. TB – tuberculosis

Close to two-thirds of countries showed a positive change in TB mortality reduction, although only 27% of them reached the WHO target of 35% reduction (**Figure 7**; Table S5 in the **Online Supplementary Document**). Among 31 countries that achieved the target, 22 (70.1%) are in the African and European WHO regions, and more than half of them are ranked in the 100 countries with the strongest health systems.

**Figure 8:**
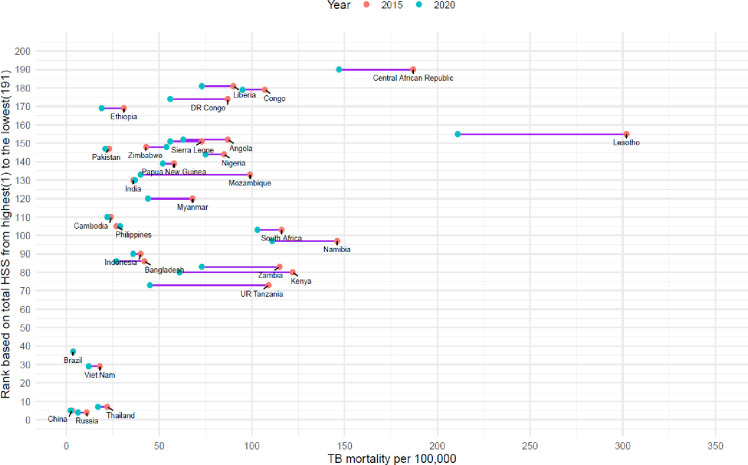
Dumbbell plot showing ranking based on total health system scores and change in TB mortality rate between 2015 and 2020 among high TB burden countries. The length of the purple line connecting the two points represents the extent of the change in TB mortality rates for each country between 2015 and 2020, expressed per 100 000 population. TB – tuberculosis.

Despite facing challenges related to weak health systems, many high TB burden countries have made substantial progress in reducing TB mortality, with 27 out of 29 (93%) countries showing positive changes in mortality reduction (**Figure 8**). Of these, seven (i.e. Vietnam, Tanzania, Mozambique, Myanmar, Kenya, Sierra Leone, and Russia) achieved a 35% reduction.

### ‘Worst’ and ‘best’ performers analysis

**Figure 9:**
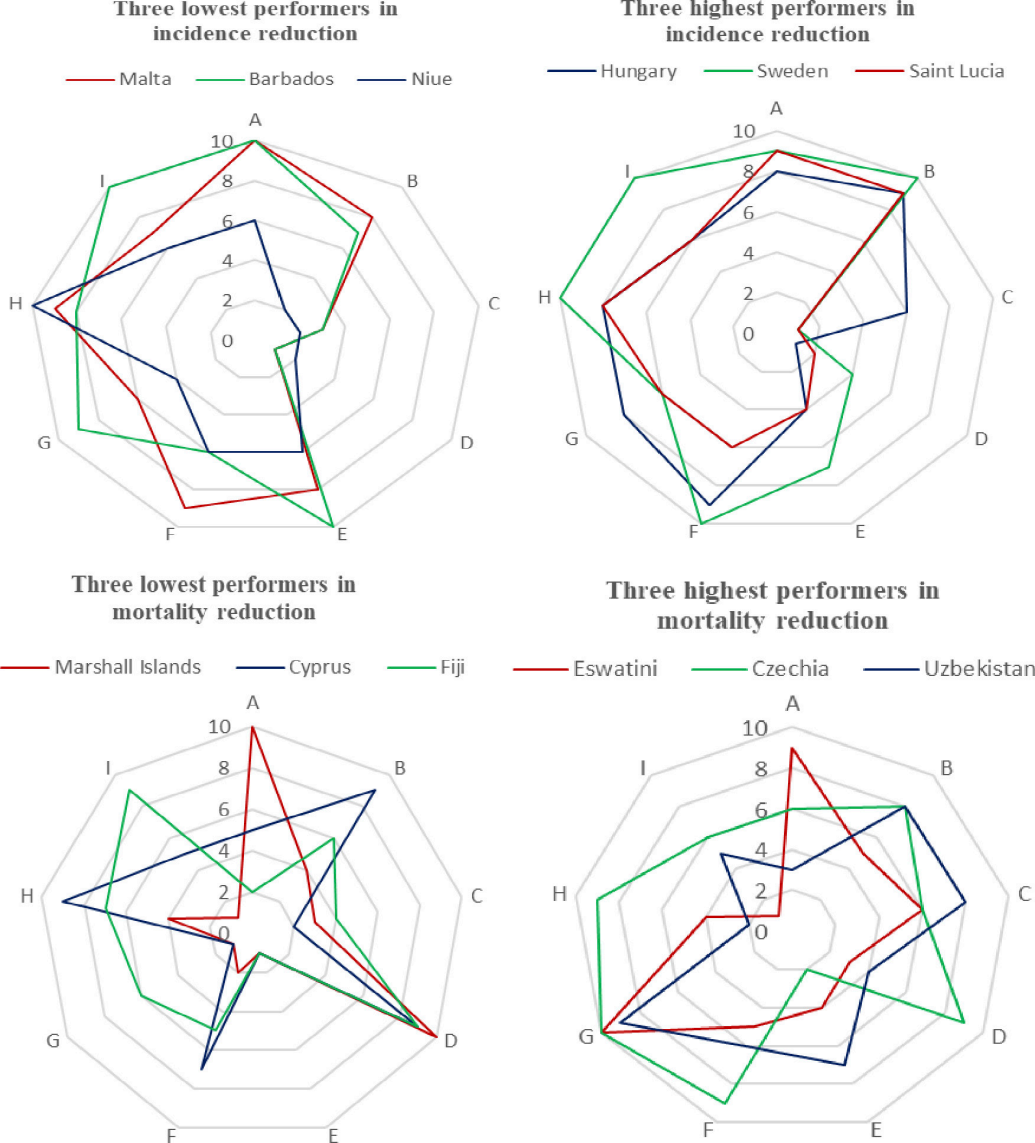
Health system dimension scores of highest and lowest performers in TB incidence and mortality reduction among low-burden countries. **Panel A.** Three lowest performers in incidence reduction. **Panel B.** Three highest performers in incidence reduction. **Panel C.** Three lowest performers in mortality reduction. **Panel D.** Three highest performers in mortality reduction. A – TB-specific financing, B – routine health service delivery, C – TB-specific health service delivery, D – general health system capacity, E – TB-specific health system capacity, F – health workforce, G – governance, H – access to medicine, I – health information system, TB – tuberculosis.

Among 162 low TB-burden countries, Malta, Barbados, and Niue recorded poor performance in TB incidence control, with incidence increasing from 2015 to 2020 by 282.7%, 100% and 65%, respectively (**Figure 9**, Panel A; Table S5 in the **Online Supplementary Document**). Niue had the weakest health system among these countries, with an overall health system score of 44 out of 90, and all three countries had relatively poor general and TB-specific health service delivery. In contrast, Hungary, Sweden, and Saint Lucia achieved a reduction of more than 54% in TB incidence, making them the top three countries in the low-burden group and globally (**Figure 9**, Panel B). Sweden, with a health system score of 67 out of 90, held the highest score among them.

Regarding mortality reduction, Marshall Islands, Cyprus, and Fiji ranked lowest among low-burden countries and globally, with TB-related mortality increasing between 2015 and 2020 by 190.9%, 83.3%, and 68.3%, respectively (**Figure 9**, Panel C). The Marshall Islands, with a health system score of 36 out of 90, had the weakest health system among these countries, and all three nations had low TB-specific health system capacity. In contrast, Eswatini, Czechia, and Uzbekistan emerged as the top three performers in TB mortality reduction, achieving more than a 50% reduction in TB mortality (**Figure 9**, Panel D). These countries have relatively strong health systems, and they are far better in general and TB-specific health system capacity and governance than the worst performers.

**Figure 10:**
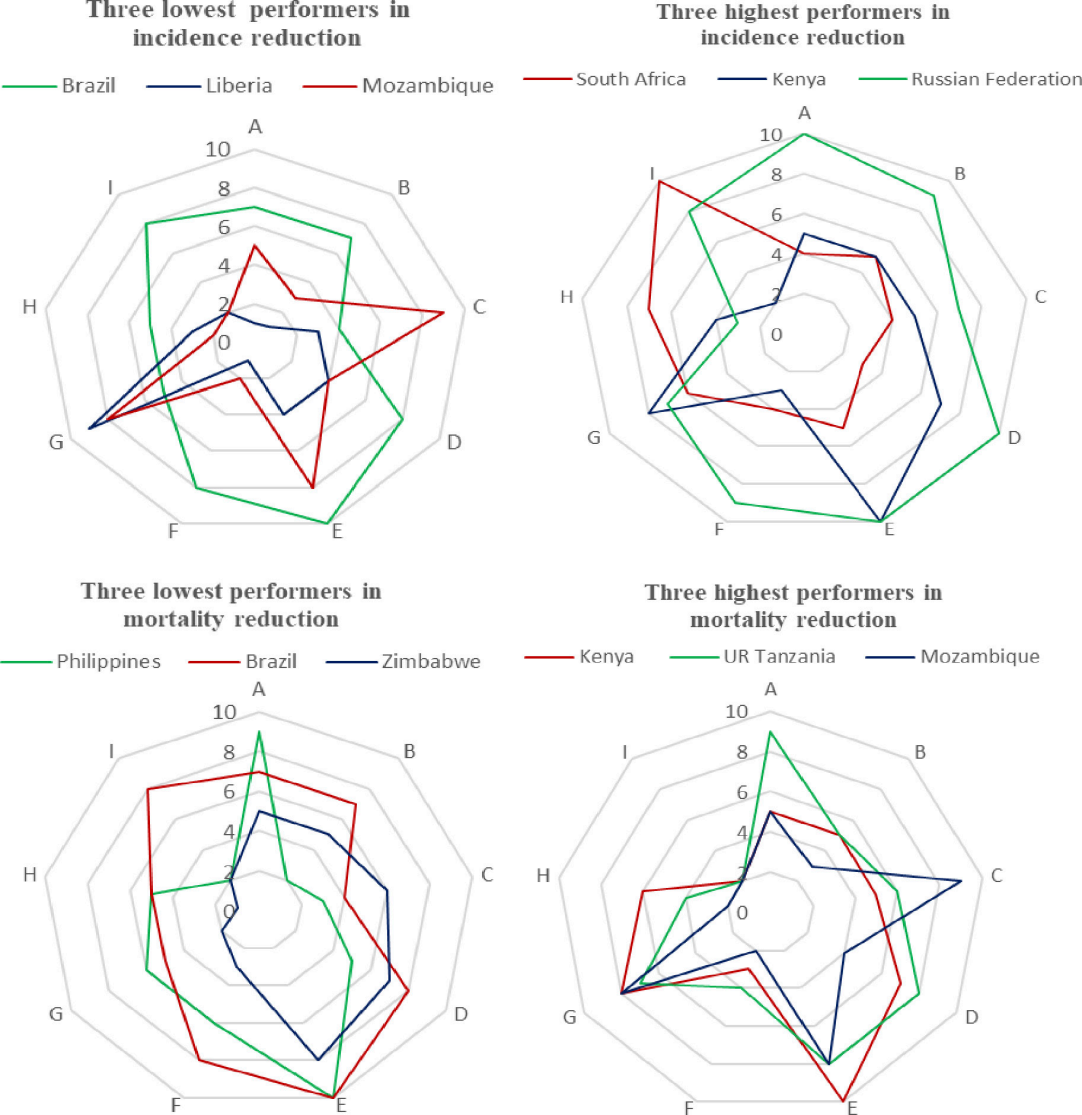
Health system dimension scores of highest and lowest performers in TB incidence and mortality reduction among high-burden countries. **Panel A.** Three lowest performers in incidence reduction. **Panel B.** Three highest performers in incidence reduction. **Panel C.** Three lowest performers in mortality reduction. **Panel D.** Three highest performers in mortality reduction. A – TB-specific financing, B – routine health service delivery, C – TB-specific health service delivery, D – general health system capacity, E – TB-specific health system capacity, F – health workforce, G – governance, H – access to medicine, I – health information system, TB – tuberculosis

Among the 29 high TB-burden countries, Brazil exhibited the poorest performance in TB incidence reduction, despite being one of the top five countries in terms of health system strength (**Figure 10**, Panel A). In contrast, South Africa, Kenya, and the Russian Federation were the top three performers, achieving reductions in TB incidence of 43.9%, 31.8%, and 31.3%, respectively (**Figure 10**, Panel B). These countries, except for Brazil, generally had stronger health systems overall and in the specific components of health information systems, access to medicine, TB financing, and health workforce (Table S5 in the **Online Supplementary Document**).

Although Mozambique struggled with TB incidence reduction, it ranked among the top three countries for TB mortality reduction among high-burden nations, alongside Kenya and the United Republic of Tanzania, all located in the African Region. These countries demonstrated strength in TB-specific health service delivery, general health system capacity, and governance compared to countries with poor performance, such as the Philippines, Brazil, and Zimbabwe (**Figure 10****,** Panels C–D).

### Factors associated with TB incidence and mortality reduction

Out of 44 identified factors, 26 unique health system and 8 environmental variables were significantly associated with TB incidence and mortality reduction in the univariable analysis. However, 10 health system and 14 environmental variables were significantly associated with incidence and mortality reduction after the models were adjusted for baseline TB burden and HDI (Table S6 in the **Online Supplementary Document**).

The final model, incorporating nine health system building blocks and three environmental characteristics, identified several associations. Annual temperature and concentration of particulate matter were negatively associated with TB incidence reduction, suggesting environmental influences on transmission (Table S7 in the **Online Supplementary Document**). Conversely, TB-specific health service delivery, TB financing, governance, and access to medicine were positively associated with TB mortality reduction. In the global model, a one-unit improvement in TB-specific service delivery increased the percentage reduction in TB mortality by 5.3% (unstandardised coefficient (*b*) *=* 5.3; 95% CI = 0.1–10.5), while a similar increase in access to medicine led to a 6.0% increase (*b* = 6.0; 95% CI = 2.3–9.7). In high TB burden countries specifically, the TB finance index had a notable impact, with a one-unit increase associated with a 10.2% greater reduction in TB mortality (*b* = 10.2; 95% CI = 3.3–23.7). These models explained a substantial portion of the variation in both TB incidence (45%) and mortality reduction (55%), highlighting their robustness in explaining outcomes within this subset (**Table 2**).

**Table 2:** Linear regression output after backward stepwise regression for the effect of the health system and environmental factors affecting TB control*

	Percent reduction in incidence†						Percent reduction in mortality†					
	**All**		**Low burden**		**High burden**		**All**		**Low burden**		**High burden**	
	***b* (95% CI)**	***P*-value**	***b* (95% CI)**	***P*-value**	***b* (95% CI)**	***P*-value**	***b* (95% CI)**	***P*-value**	***b* (95% CI)**	***P*-value**	***b* (95% CI)**	***P*-value**
**Health system factors**												
TB specific financing	−5.5 (−12.0, 0.9)	0.09	−5.0 (−12.2, 2.2)	0.16	NS		NS		NS		10.2 (3.3, 23.7)	<0.05
Routine health service delivery	NS		NS		2.1 (−1.4, 5.5)	0.08	NS		−3.6 (−7.9, 0.7)	0.34	5.9 (3.3, 11.5)	<0.01
TB health service delivery	NS		NS		NS		5.3 (0.1, 10.5)	<0.05	6.5 (0.5, 12.5)	<0.05	NS	
General health system capacity	NS		NS		NS		−5.8 (−12.6, 1.0)	0.32	−5.8 (−13.2, 1.5)	0.21	NS	
TB-specific health system capacity	NS		−5.8 (−13.5, 1.8)	0.44	NS		NS		NS		NS	
Health workforce	NS		NS		−9.2 (−19.1, 0.6)	0.06	NS		NS		NS	
Governance	NS		NS		NS		4.2 (1.5, 6.9)	<0.01	4.9 (1.8, 8.0)	<0.05	NS	
Access to medicine	NS		NS		NS		6.0 (2.3, 9.7)	<0.01	5.5 (1.3, 9.7)	<0.01	7.3 (1.4, 13.0)	<0.05
Health information system	−4.7 (−9.6, 0.09)	0.29	−5.5 (−11.1, 0.1)	0.48	NS		NS		NS		NS	
**Environmental factors**												
Average temperature	−1.4 (−2.1, −0.7)	<0.001	−1.4 (−2.2, −0.6)	<0.001	−0.6 (−1.4, 0.3)	0.33	−0.7 (−1.3, −0.1)	<0.05	−0.7 (−1.4, 0.6)	0.63	−0.7 (−1.6, 0.2)	0.55
Rainfall	NS		NS		−0.005 (−0.01, 0.001)	0.51	−0.004 (−0.009, 0.001)	0.22	−0.004 (−0.01, 0.008)	0.43	NS	
Particulate matter	NS		NS		−0.4 (−0.7, −0.08)	<0.05	NS		NS		−0.3 (−0.6, 0.05)	0.09
R^2^ of the model	0.10		0.11		0.45		0.24		0.20		0.55	
AIC	1316.6		1139.5		135.6		1260		1082.9		148.2	

AIC – Akaike information criterion, *b* – unstandardised coefficient, CI – confidence interval, NS – not selected, R^2^ – coefficient of determination, TB – tuberculosis

*All models were adjusted for total current health expenditure as a percentage of GDP and HDI of countries in 2105, with the model for all 191 countries having an additional adjustment for TB burden; the low burden model (for 162 countries) was classified as having a low TB burden, and high burden model (for 29 countries) was classified as having high TB burden by WHO in 2015.

†The regression used *P* > 0.2 to exclude and *P* < 0.10 to include variables.

## DISCUSSION

This study is the first to quantify the impact of the health system and environmental factors on global progress towards the WHO End TB strategy milestones. Globally, TB mortality declined by 9.2% and TB incidence declined by 11% between 2015 and 2020, missing the first milestone. Significant variability was observed at national and regional levels in achieving these milestones, as well as in rates of TB mortality and incidence reduction. These inequalities were associated with differences in multiple health systems and environmental factors, including TB financing, service delivery, access to medicine, health system governance, average annual temperature, and air particulate matter concentration.

High-burden countries, accounting for 87% of new TB cases globally [4], showed good progress in reducing mortality, with an average reduction of 24.2%. However, these countries are only halfway to meeting the 2020 target for incidence reduction, likely due to relatively weak health systems. Among them, Brazil showed the poorest performance in TB incidence reduction. Despite having a good overall health system, Brazil faces challenges in reaching vulnerable populations [34]. Socioeconomic inequalities, urbanisation, and overcrowded living conditions further contribute to TB transmission in the country [35]. In contrast, Kenya was one of the top-performing high-burden countries, achieving significant incidence and mortality reductions. The country has strengthened its TB control programme through extensive community health worker networks and improved diagnostic services [36]. Strengthening health systems in high-burden countries should be prioritised to maximise progress and achieve the WHO end TB strategy targets.

The WHO European Region showed significant progress in reducing TB incidence and mortality [6], possibly due to its strong health systems. Slovakia, Norway, Hungary, and Sweden achieved more than a 50% reduction in TB incidence. while Russia, Malta, Uzbekistan, and Czechia contributed the most to overall mortality reduction. According to the WHO regional report, the European Region has the strongest public health responses in surveillance, monitoring, emergency planning, immunisation, environmental health, and health protection [37], which are key for the effective implementation of the End TB strategy. The African Region also made notable progress in TB mortality reduction, achieving an 18% decrease, over halfway to the 2020 milestone; here, Tanzania, Eswatini, and Mozambique were the top contributors to overall mortality reduction. While the African Region has weaker health systems compared to other regions, as shown by a previous review [38], several factors may have contributed to this progress. These include the universal Bacillus Calmette-Guérin vaccination programme implemented across the region [39], the established drug-resistant TB treatment programmes, the introduction of more effective short-course treatments, and significant success in addressing TB/HIV co-infection [40,41].

Reduction of TB mortality was positively associated with access to medicine across all burden categories. Countries with high mortality reduction, such as Tanzania, Czechia, Eswatini, and Uzbekistan, generally had better access to medicines compared to those with poorer performance. Without an effective TB vaccine for adults, the basic strategy to combat TB has focussed on improving diagnosis and treatment access for individuals who are ill and seek care at a health facility [42]. This includes expanding access to new, effective drugs and regimens, such as TB preventive treatment [43]. The premise is that curing patients with active disease eliminates mortality and reduces disease prevalence by reducing transmission [44]. Improved access to medicine was also found to be a successful approach in global and regional infectious disease control programmes for malaria [45,46] and HIV [47]. To sustain progress, countries should improve access to medicine for TB patients and strictly follow the WHO-endorsed directly observed treatment, short course and short course-plus programmes, which include an uninterrupted supply of all essential anti-TB drugs [48].

Strong health system governance was positively associated with TB mortality reduction, making it a critical, but often neglected aspect of national TB programmes. Governance determines the effective and efficient implementation of the programme at both national and peripheral levels, involving individuals, the TB community, civil society, and subnational government entities [49,50]. Countries such as Denmark, New Zealand, and Belgium, which have strong governance, have seen notable successes in reducing TB mortality. A UN high-level meeting on the fight against TB recommended all countries, especially high-burden ones, ensure high-level multisectoral collaboration and accountability, led by heads of state or government, including regular progress reviews on TB control to achieve the WHO End TB targets [43]. Therefore, countries must adhere to essential governance benchmarks for National TB Programmes (i.e. transparency, inclusiveness, legal frameworks, and process efficiency and effectiveness) [49]. Political stability is also critical for creating an environment that supports sustainable TB control efforts, enhances resource allocation, and promotes accountability in programme implementation.

Reducing TB mortality was positively associated with better TB-specific health service delivery. To minimise mortality caused by TB, it is essential that everyone who develops the disease can quickly access diagnosis and treatment, as 70% of untreated smear-positive TB cases will die within 10 years of their diagnosis [51]. Providing TB prevention, diagnosis, and treatment services within the broader context of progress towards universal health coverage is also a key component of the WHO End TB strategy [7]. Therefore, to achieve global mortality reduction targets by 2035, countries should strive to improve TB case detection and treatment rates. They should also comply with new WHO consolidated guidelines on TB preventive therapy [52], diagnosis [53], and TB disease treatment [54,55].

Additionally, TB mortality reduction was associated with TB financing and routine health service delivery among high TB burden countries. This is consistent with the Global Fund’s current focus on routine service delivery, including investment in quality improvement [56]. Progress in reducing the burden of TB disease also requires adequate funding sustained over many years [7]. The UN recommends increasing international donor and domestic funding for the TB response, from both existing or new innovative mechanisms, so that funding levels are proportional to the burden of disease [43]. A recent global plan for 2023–30 also estimates much higher funding needs of USD 15–32 billion per year in low- and middle-income countries [57]. International donor funding only accounted for 53% of the financing available in the 26 high-burden and two global TB watchlist countries (i.e. Cambodia and Zimbabwe), and it covers less than 40% of the total funding required for the implementation of national strategic plans for TB [58]. Therefore, improving domestic funding to combat TB, especially in middle-income countries with a high TB burden, is key to reaching global TB targets, as recommended by the UN high-level meeting on TB [43].

Of the environmental factors, higher temperatures were found to negatively impact TB incidence and mortality reduction, which is well aligned with previous studies [20,59–61]. In addition, higher air particulate matter concentrations showed a negative association with TB incidence reduction in high-burden countries, which is also supported by two studies in China [62,63]. Particulate matter is a key indicator of air pollution and is directly associated with damage to the lungs and the respiratory system [64]. It could also directly impair or modify the immunological response of the human respiratory system by inducing oxidative and nitrosative stressors [65,66], and by weakening alveolar macrophage activity and mucociliary clearance, which are vital defence mechanisms against TB [67,68]. This increases the host's susceptibility to TB disease. Therefore, it is important to integrate responses to environmental conditions into national TB research agendas, especially in high-burden countries [69]. For example, governments and civil society should follow WHO air-quality guidelines to reduce human exposure to air pollution and its adverse effects [70]. In addition, countries should work to rapidly reduce carbon dioxide and other greenhouse gas emissions to limit the global temperature increase [71]. It is also important to scale up public health interventions to mitigate the effect of climate change, as recommended by Centres for Disease Control and Prevention [22].

This study has some limitations. We chose this ecological study design as the data for both the outcome variables and predictors were only available at the country level, and conducting an individual-level study would have been impractical due to the scale and nature of the data required. The design may have introduced ecological fallacy, as the country level analysis and the associations may not necessarily hold at the individual level. As the data for most health system indicator variables were not available for all years, it was not possible to conduct time-series analyses for the factors in each year, so we used average values. This may not have captured changes in TB reporting methods, guidelines, and policies through time. In addition, shifts in global health priorities and funding caused by COVID-19 during our study period could have affected TB incidence and mortality rates. Furthermore, the WHO building blocks we used are organised around a supply model that features detailed aspects of service delivery, but does not consider demand-side aspects. As the data were not available, we did not include social mobilisation activities, which can be a critical component of health systems in low- and middle-income countries [72]. Moreover, important environmental variables such as hours of sunshine, wind speed, and atmospheric pressure were not included since the data were not available for all countries. Therefore, further studies using complete health system data that consider demand aspects of the health system can help generate better evidence on the health system readiness for implementing disease control programmes like the End TB strategy. We suggest further studies at a lower geographical scale (i.e. districts) to check if there are spatial variations within countries in achieving the end-TB targets.

## CONCLUSIONS

Progress towards achieving the WHO End TB milestones varies and is partially influenced by the strength of national health systems and environmental conditions. Weak health systems are prevalent in most high-burden countries. Therefore, strengthening health systems, specifically in TB financing, service delivery, and access to medicine, should be prioritised to meet global TB mortality reduction targets. Additionally, we also highlighted the importance of improving health system governance in all countries to enhance TB control efforts. Environmental factors, such as average temperature and particulate matter concentration, were found to negatively impact TB incidence reduction. Therefore, countries should follow WHO’s air quality guidelines and urgently reduce carbon dioxide and other greenhouse gas emissions to minimise the environmental impact on TB control.

## Additional material


Online Supplementary Document
