# Supplementary material for: Health system and environmental factors affecting global progress towards achieving End TB targets between 2015 and 2020
Source: J Glob Health. 2025 Jan 10;15:04004. doi: 10.7189/jogh.15.04004 (PMC11719748; doi:10.7189/jogh.15.04004)
Supplement: Online Supplementary Document [file jogh-15-04004-s001.pdf]

Supplement to: Wolde HF, Clements ACA, Alene KA. Health system and environmental factors affecting global progress towards achieving End TB targets between 2015 and 2020. J Glob Health. 2025;15:04004.

Contents

Table S1: List of variables extracted with data source and variable coverage....................................... 2

Table S2: Variables included in the final analysis and their definition 5

Table S3: Relevance of variables included in the model to TB control.. 11

Table S4: Summary of environmental factors and principal component analysis (PCA) results 11

Environmental characteristics 11

Dimension reduction using PCA.. 12

Variables contribution for each principal factor 13

Table S5: Health System Scores 15

Table S6: Univariable and adjusted effect of health system and environmental factors on percent reduction of TB incidence and mortality 20

Table S7: Linier and logistic regression outputs on the effect of health system building blocks on TB control. 25

Additional figures 27

Table S1: List of variables extracted with data source and variable coverage.

We exclusively used data from well-established and reputable sources, which are known for are known for their rigorous data collection, validation, and reporting procedures. The WHO Global TB Databases provides detailed methodologies for data collection, validation, and reporting of TB statistics. These datasets undergo thorough verification processes to ensure consistency and accuracy across countries. The World Bank and UNDP also utilizes standardized surveys and data collection methodologies, ensuring comparability across countries. We also cross-verified data points from multiple sources to ensure consistency.

**Variables**	**Indicators**	**Percentage of available data**		**Data source**	**Remark**
		2015	*2020*		
Outcome variables					
TB incidence	TB incidence (cases per 100,000 population)	100%	100%	WHO-TB program(1)	Included as outcome variable
TB mortality	TB mortality (deaths per 100,000 population)	100%	100%	WHO-TB program(1)	Included as outcome variable
Control variables: *only baseline (2015) data is extracted for all control variables*					
Baseline Human Development index	Human Development Index	100%	NA	UNDP (2)	Included as control variable
Baseline TB burden	TB burden category	100%	NA	WHO(3)	Included as control variable
Baseline health financing(general)	Domestic general government health expenditure (% of current health expenditure)	96.4%	NA	WDI(4)	Included as control variable
	Out-of-pocket expenditure per capita (current US$)	96.4%	NA	WDI(4)	Included as control variable
	Current health expenditure (% of GDP)	96.4%	NA	WDI(4)	Included as control variable
	External health expenditure (% of current health expenditure)	86.08	NA	WDI(4)	Included as control variable
	Domestic private health expenditure (% of current health expenditure)	95.4%	NA	WDI(4)	Included as control variable
	Out-of-pocket expenditure (% of current health expenditure)	95.9%	NA	WDI(4)	Included as control variable

	Current health expenditure per capita (current US$)	95.9%	NA	WDI(4)	Included as control variable
**Health system factors**					
TB specific financing	Average cost of drugs budgeted per patient for DSTB treatment	56.7%	59.8%	WHO-TB program(1)	Included as a factor
	Average cost of drugs budgeted per patient for MDR-TB treatment	57.7%	59.8%	WHO-TB program(1)	Included as a factor
	Average cost of drugs budgeted per patient for pre-XDR/XDR-TB	50%	54.1%	WHO-TB program(1)	Included as a factor
	Average cost of drugs budgeted per patient for TB preventive treatment	NA	51.5%	WHO-TB program(1)	End line data included as a factor
	Budget gap for TB	71.6%	71.6%	WHO-TB program(1)	Included as a factor
	Total actual expenditure for TB	69.6%	67.5%	WHO-TB program(1)	Included as a factor
Routine health service delivery	Measles immunization coverage	99%	96.9%	HNP(5)	Included as a factor
	ART coverage	71.6%	68.6%	HNP(5)	Included as a factor
	DPT3 immunization coverage	100%	98.5%	HNP(5)	Included as a factor
	Universal health coverage index	99%	99%	HNP(5)	Included as a factor
	Four ANC visit	11.9%	7.7%	HNP(5)	Excluded due to missing data
	Births attended by skilled birth attendant	49%	20.6%	HNP(5)	Excluded due to missing data
TB health service delivery	TB case detection rate	95.4%	94.3%	HNP(5)	Included as a factor
	TB treatment success rate	91.2%	92.8%	HNP(5)	Included as a factor
	BCG immunization coverage	80.4%	78.4%	HNP(5)	Included as a factor
Health system capacity and workforce					
General health system capacity	Hospitals per 100,000 population	70.1%	NA	WHO-GHO(6)	Base line data included as a factor
	Health centres per 100,000 population	57.2%	NA	WHO-GHO(6)	Base line data included as a factor
	Health posts per 100,000 population	57.2%	NA	WHO-GHO(6)	Base line data included as a factor
	Hospital beds per 10000 population	52.1%	18.6%	WHO-GHO(6)	Base line data included as a factor
TB specific health system capacity	Number of TB basic management units	56.2%	54.6%	WHO-TB program(1)	Included as a factor

	Number of TB diagnostic sites	84%	83.5%	WHO-TB program(1)	Included as a factor
Health work force	Physicians per 1,000 population	54.6%	21.13%	HNP(5)	Base line data included as a factor
	Nurses or midwifes per 1,000 population	64.9%	27.8%	HNP(5)	Base line data included as a factor
	CHW per 1,000 population	2.1%	NA	HNP(5)	Excluded due to missing data
Governance	Logistic performance score	81.4%	80.9%	LPI(7)	Included as a factor
	Control of corruption index	97.9%	97.9%	WGI(8)	Included as a factor
	Rule of low index	96.9%	97.9%	WGI(8)	Included as a factor
	Government effectiveness index	97.9%	97.9%	WGI(8)	Included as a factor
	Political stability index	99%	100%	WGI(8)	Included as a factor
	Regulatory quality index	97.9%	97.9%	WGI(8)	Included as a factor
	Voice and accountability index	99%	100%	WGI(8)	Included as a factor
	Policy and action plan for CRD	88.7%	NA	WHO-GHO(6)	Baseline data Included as a factor
Access to medicine	Availability of medicines in public facilities	18.04%	NA	WHO-GHO(6)	Excluded due to missing data
	Children treated for ARI	29.4%	NA	DHS(9)	Excluded due to missing data
	Children treated with antibiotics for fever	20.1%	NA	DHS(9)	Excluded due to missing data
	Children treated with oral rehydration therapy	50.5%	NA	WHO-GHO(6)	Included as a factor
	Children care seeking	34.5%	NA	WHO-GHO(6)	Excluded due to missing data
	Pregnant women treated for syphilis	52.1%	45.9%	WHO-GHO(6)	Baseline data included as a factor
Health information system	Completeness of birth registration	10.3%	3.1%	HNP(5)	Excluded due to missing data
	Death cause registration	29.4%	NA	WHO-GHO(6)	Imputed data included as a factor
	EHR in the country	62.9%	NA	WHO-GHO(6)	Included as a factor
	Completeness of death cause registration	60.8%	NA	WHO-GHO(6)	Included as a factor
Environmental factors	Particulate matter	100%	100%	WHO-GHO(6)	Included as a factor
	Mean temperature	100%	100%	CCKP(10)	Included as a factor
	Rainfall (precipitation)	100%	100%	CCKP(10)	Included as a factor

ANC: Antenatal Care, ART: Anti-retroviral Treatment, ARI: Acute Respiratory Infection, BCG: Bacillus Calmette–Guérin, CHW: Community Health Worker, CRD: Chronic Respiratory Disease, DSTB: Drug Susceptible Tuberculosis, EHR: Electronic health record, MDRTB: Multi-Drug Resistant Tuberculosis, TB: Tuberculosis, XDR-TB: Extremely Drug Resistant Tuberculosis

Variables with < 50% data are excluded.

References

1. World health organization global TB program available. Available at: Global Tuberculosis Programme (who.int) [Cited 15th December 2022].
2. Human Development Index(HDI) United Nations Development Programme. Available at: https://hdr.undp.org/data-center/human-development-index#/indicies/HDI. Access on August 10 2022 [Internet].
3. World Health Organization. Global tuberculosis report. Geneva, Switzerland, 2016. Available at https://www.who.int/publications/i/item/9789241565394 [Cited 5th May 2023].
4. World Bank. World Development Indicators database. Available at: https://databank.worldbank.org/data/source/world-development-indicators [Cited 15th December 2022].
5. World Bank. Health Nutrition and Population Statistics. Available at: https://databank.worldbank.org/data/source/health-nutrition-and-population-statistics [Cited 15th December 2022].
6. World Health Organization. Global Health Observatory. Available at: https://www.who.int/data/gho/data/indicators/indicators-index [Cited 15th December 2022.
7. World Bank. Logistics Performance Index. Available at: https://lpi.worldbank.org/ [Cited 15th December 2022].
8. World Bank. Worldwide Governance Indicators database. Available at: https://databank.worldbank.org/data/source/worldwide-governance-indicators. [Cited 15th December 2022].
9. United States Agency for International Development. Demographic Health Survey. Available at: http://www.statcompiler.com [Cited 15th December 2022].
10. World Bank Group, Climate Change Knowledge Portal. Available at: https://climateknowledgeportal.worldbank.org/download-data.

Table S2: Variables included in the final analysis and their definition.

Variables	Indicators	Definition
Outcome	TB incidence (cases per 100,000 population)	New and relapse TB cases arising in a given year, expressed as the rate per 100 000 population. All forms of TB are included, including cases in people living with HIV. Published values are rounded to three significant figures (1).
	TB mortality (deaths per 100,000 population)	The estimated number of deaths from all forms of TB, expressed as the rate per 100 000 population (1).

Control (All estimates are for 2015)	Human Development Index	It is a summary measure of average achievement in key dimensions of human development: a long and healthy life, being knowledgeable and have a decent standard of living. The HDI is the geometric mean of normalized indices for each of the three dimensions (2).
	TB burden	TB burden is classified as high and low based on the WHO 2016 global TB report which showed the list of 30 high TB burden countries based on the estimate(data) from all countries in 2015(3)
	Domestic general government health expenditure (% of current health expenditure)	Share of current health expenditures funded from domestic public sources for health. Domestic public sources include domestic revenue as internal transfers and grants, transfers, subsidies to voluntary health insurance beneficiaries, non-profit institutions serving households (NPISH or enterprise financing schemes as well as compulsory prepayment and social health insurance contributions. They do not include external resources spent by government on health (4)
	Out-of-pocket expenditure per capita (current US$)	Health expenditure through out-of-pocket payments per capita in USD. Out of pocket payments are spending on health directly out of pocket by households in each country (4)
	Current health expenditure (% of GDP)	Level of Current health expenditure expressed as a percentage of GDP. Estimates of current health expenditure include health care goods and services consumed during each year. This indicator does not include capital health expenditures such as buildings, machinery, IT and stocks of vaccines for emergency or outbreaks (4)
	External health expenditure (% of current health expenditure)	Share of current health expenditures funded from external sources. External sources compose of direct foreign transfers and foreign transfers distributed by government encompassing all financial inflows into the national health system from outside the country. External sources either flow through the government scheme or are channelled through non-governmental organizations or other schemes (4)
	Domestic private health expenditure (% of current health expenditure)	Share of current health expenditures funded from domestic private sources. Domestic private sources include funds from households, corporations, and non-profit organizations. Such expenditures can be either prepaid to voluntary health insurance or paid directly to healthcare providers (4).
	Out-of-pocket expenditure (% of current health expenditure)	Share of out-of-pocket payments of total current health expenditures. Out-of-pocket payments are spending on health directly out-of-pocket by households (4)
	Current health expenditure per capita (current US$)	Current expenditures on health per capita in current US dollars. Estimates of current health expenditures include health care goods and services consumed during each year (4)
TB specific financing(factor)	Average cost of drugs budgeted per patient for DSTB treatment	Average cost of drugs budgeted per patient for drug-susceptible TB treatment, excluding buffer stock (US Dollars) (1)
	Average cost of drugs budgeted per patient for MDR-TB treatment	Average cost of drugs budgeted per patient for MDR-TB treatment, excluding buffer stock (US Dollars) (1)

	Average cost of drugs budgeted per patient for pre-XDR/XDR-TB	Average cost of drugs budgeted per patient for pre-XDR/XDR-TB treatment, excluding buffer stock (US Dollars) (1)
	Average cost of drugs budgeted per patient for TB preventive treatment	Average cost of drugs budgeted per patient for TB preventive treatment, excluding buffer stock (US Dollars) (1)
	Budget gap for TB	This is the difference between the total budget required for TB in USD and total expected funding for all budget line items (US Dollars). The total budget required and expected funding are the sum of budget for drugs to treat drug-susceptible TB , laboratory infrastructure, equipment and supplies , programme costs to treat drug-resistant TB, operational research and surveys, all other budget line items, required patient support, programme costs to treat drug-susceptible TB, drugs to treat drug-resistant TB, National TB Programme staff (central unit staff and subnational TB staff) , collaborative TB/HIV activities , and TB preventive treatment: drugs (1)
	Total actual expenditure for TB	This variable is the sum of countries actual expenditure on drugs to treat DSTB, laboratory infrastructure, equipment and supplies, program costs to treat DRTB, operational research and survey, patient support, program costs to treat DSTB, drugs to treat DRTB, NTP staff, collaborative TB/HIV activities and all other budget line items (1)
Routine health service delivery (factor)	Measles immunization coverage	Child immunization, measles, measures the percentage of children ages 12-23 months who received the measles vaccination before 12 months or at any time before the survey. A child is considered adequately immunized against measles after receiving one dose of vaccine. It estimated based on WHO and UNICEF estimates(5).
	ART coverage	Antiretroviral therapy coverage indicates the percentage of all people living with HIV who are receiving antiretroviral therapy and it is estimated based on UNAIDS estimate(5).
	DPT3 immunization coverage	Child immunization, DPT, measures the percentage of children ages 12-23 months who received DPT vaccinations before 12 months or at any time before the survey. A child is considered adequately immunized against diphtheria, pertussis (or whooping cough), and tetanus (DPT) after receiving three doses of vaccine(5).
	Universal health coverage index	Coverage index for essential health services (based on tracer interventions that include reproductive, maternal, newborn and child health, infectious diseases, noncommunicable diseases and service capacity and access). It is presented on a scale of 0 to 100(5).
TB health service delivery(factor)	TB case detection rate	Tuberculosis case detection rate (all forms) is the number of new and relapse tuberculosis cases notified to WHO in a given year, divided by WHO's estimate of the number of incident tuberculosis cases for the same year, expressed as a percentage. Estimates for all years are recalculated as new information becomes available and techniques are refined, so they may differ from those published previously(5).

	TB treatment success rate	Tuberculosis treatment success rate is the percentage of all new tuberculosis cases (or new and relapse cases for some countries) registered under a national tuberculosis control programme in a given year that successfully completed treatment, with or without bacteriological evidence of success ("cured" and "treatment completed" respectively) (5).
	BCG immunization coverage	Child immunization rate, BCG is the percentage of children ages 12-23 months who received vaccinations before 12 months or at any time before the survey for BCG. A child is considered adequately immunized after one dose. WHO and UNICEF estimates are taken in this case (5)
General health system capacity (factor)	Hospital per 100000 population	Number of hospitals, including the following hospital categories: rural and district, provincial (second level referral), regional/specialized/teaching and research hospitals (tertiary care), from the public and private sectors, per 100,000 population (6)
	Health centre per 100000 population	Number of health centres from the public and private sectors, per 100,000 population(6).
	Health post per 100000 population	Number of health posts from the public and private sectors, per 100,000 population. Health posts are either community centres or health environments with a very limited number of beds with limited curative and preventive care resources normally assisted by health workers or nurses(6).
	Hospital bed per 10000 population	Hospital beds include inpatient beds available in public, private, general, and specialized hospitals, and rehabilitation centres. In most cases beds for both acute and chronic care are included (5).
TB specific health system capacity (factor)	Number of TB basic management units	The number of TB Basic Management Units in the country at the end of the reporting year(1).
	Number of TB diagnostic sites	The number of sites providing TB diagnostic services using smear microscopy (including fluorescent) at the end of the reporting year(1).
Health work force (factor)	Physician per 1000 population	Physicians include generalist and specialist medical practitioners, per 100,000 population(5).
	Nurse or midwife per 1000 population	Nurses and midwives include professional nurses, professional midwives, auxiliary nurses, auxiliary midwives, enrolled nurses, enrolled midwives and other associated personnel, such as dental nurses and primary care nurses, per 1000 population(5).
Governance (factor)	Logistic performance index (LPI)	The LPI is an interactive benchmarking tool created to help countries identify the challenges and opportunities they face in their performance on trade logistics and what they can do to improve their performance(7)
	Control of corruption index	Perceptions of the extent to which public power is exercised for private gain, including both petty and grand forms of corruption, as well as "capture" of the state by elites and private interests. Estimate gives the country's score on the aggregate indicator, in units of a standard normal distribution, i.e. ranging from approximately -2.5 to 2.5(8).
	Rule of low index	Perceptions of the extent to which agents have confidence in and abide by the rules of society, and in particular the quality of contract enforcement, property rights, the police, and the courts, as well as the likelihood of crime and violence. Estimate gives the country's score on the aggregate indicator, in units of a standard normal distribution, i.e. ranging from approximately -2.5 to 2.5(8).
	Government effectiveness index	Perceptions of the quality of public services, the quality of the civil service and the degree of its independence from political pressures, the quality of policy formulation and implementation, and the credibility of the government's commitment to such policies. Estimate gives the country's score on

		the aggregate indicator, in units of a standard normal distribution, i.e. ranging from approximately -2.5 to 2.5(8).
	Political stability index	Political Stability and Absence of Violence/Terrorism measures perceptions of the likelihood of political instability and/or politically motivated violence, including terrorism. Estimate gives the country's score on the aggregate indicator, in units of a standard normal distribution, i.e. ranging from approximately -2.5 to 2.5(8).
	Regulatory quality index	Perceptions of the ability of the government to formulate and implement sound policies and regulations that permit and promote private sector development. Estimate gives the country's score on the aggregate indicator, in units of a standard normal distribution, i.e. ranging from approximately -2.5 to 2.5(8).
	Voice and accountability index	Perceptions of the extent to which a country's citizens are able to participate in selecting their government, as well as freedom of expression, freedom of association, and a free media. Estimate gives the country's score on the aggregate indicator, in units of a standard normal distribution, i.e., ranging from approximately -2.5 to 2.5(8).
	Policy and action plan for CRD	Indicates whether or not the country has an operational policy, strategy or action plan for chronic respiratory diseases (6).
Health information system (factor)	Completeness of death cause registration	Completeness of estimated level of coverage of deaths that are registered with cause-of-death information (6).
	EHR in the country	Existence of a national electronic health record (EHR). Electronic health records (EHRs) are real-time, patient-centred records that provide immediate and secure information to authorised users. EHRs typically contain a patient's medical history, diagnoses and treatment, medications, allergies, immunizations, as well as radiology images and laboratory results. A National Electronic Health Records system is most-often implemented under the responsibility of the national health authority and will typically make a patient's medical history available to health professionals in health care institutions and provide linkages to related services such as pharmacies, laboratories, specialists, and emergency and medical imaging facilities(6).
	Ill-defined causes in cause-of-death registration (%)	The percentage of total deaths that has been assigned to ill-defined causes as reported to WHO (6).
Access to medicine(factor)	Availability of medicines in public facilities	Median availability of selected generic medicines in public health facilities (6)
	Children treated for ARI	Percentage of children with symptoms of ARI who sought treatment/advice from public sector facility(9)
	Children treated with antibiotics for fever	Percentage of children who took antibiotic drugs for fever from recent DHS surveys(9)
	Pregnant women treated for syphilis	Percentage of antenatal care attendees positive for syphilis who received treatment. Numerator: Number of antenatal care attendees with a positive syphilis serology who received at least one dose of benzathine penicillin 2.4 mU IM. Denominator: Number of antenatal care attendees with a positive syphilis serology(6).
	Children treated with oral rehydration therapy	Percentage of children aged 0–59 months who had diarrhoea in the two weeks prior to the survey and received oral rehydration salts. Numerator: Number of children aged 0–59 months with diarrhoea in

		the two weeks prior to the survey receiving oral rehydration salts. Denominator: Total number of children aged 0–59 months with diarrhoea in the two weeks prior to the survey(6).
Environmental factors	Particulate matter	The mean annual concentration of fine suspended particles of less than 2.5 microns in diameters is a common measure of air pollution(6).
	Mean temperature	Average annual temperature of countries obtained from Climatic Research Unit gridded Time Series (CRU TS) dataset which provides quality-controlled values from thousands of weather stations worldwide(10).
	Rainfall (precipitation)	Sum of the precipitation values over identified year obtained from Climatic Research Unit gridded Time Series (CRU TS) dataset which provides quality-controlled values from thousands of weather stations worldwide(10).

ANC: Antenatal Care, ART: Anti-retroviral Treatment, ARI: Acute Respiratory Infection, BCG: Bacillus Calmette–Guérin, CHW: Community Health Worker, CRD: Chronic Respiratory Disease, DSTB: Drug Susceptible Tuberculosis, EHR: Electronic health record, MDRTB: Multi-Drug Resistant Tuberculosis, TB: Tuberculosis, XDR-TB: Extremely Drug Resistant Tuberculosis

References

1. World health organization global TB program available. Available at: Global Tuberculosis Programme (who.int) [Cited 15th December 2022].
2. Human Development Index(HDI) United Nations Development Programme. Available at: https://hdr.undp.org/data-center/human-development-index#/indicies/HDI. Access on August 10 2022 [Internet].
3. World Health Organization. Global tuberculosis report. Geneva, Switzerland, 2016. Available at https://www.who.int/publications/i/item/9789241565394 [Cited 5th May 2023].
4. World Bank. World Development Indicators database. Available at: https://databank.worldbank.org/data/source/world-development-indicators [Cited 15th December 2022].
5. World Bank. Health Nutrition and Population Statistics. Available at: https://databank.worldbank.org/data/source/health-nutrition-and-population-statistics [Cited 15th December 2022].
6. World Health Organization. Global Health Observatory. Available at: https://www.who.int/data/gho/data/indicators/indicators-index [Cited 15th December 2022.
7. World Bank. Logistics Performance Index. Available at: https://lpi.worldbank.org/ [Cited 15th December 2022].
8. World Bank. Worldwide Governance Indicators database. Available at: https://databank.worldbank.org/data/source/worldwide-governance-indicators. [Cited 15th December 2022].
9. United States Agency for International Development. Demographic Health Survey. Available at: http://www.statcompiler.com [Cited 15th December 2022].
10. World Bank Group, Climate Change Knowledge Portal. Available at: https://climateknowledgeportal.worldbank.org/download-data.

Table S3: Relevance of variables included in the model to TB control

Variable	Relevance for TB control
**TB specific financing**	Adequate funding is essential for sustaining TB control programs, ensuring the availability of diagnostics, treatment, and support services. It impacts the overall ability to implement effective TB interventions and maintain the infrastructure needed for TB control
**Routine health service delivery**	Strong routine health services are necessary for identifying and managing TB cases. Integrated health services can help in early diagnosis, treatment, and follow-up, which are crucial for reducing TB incidence and mortality.
**TB health service delivery**	Specialized TB services ensure that patients receive appropriate care, including accurate diagnosis, effective treatment, and management of drug-resistant TB. This directly influences the success of TB control measures.
**General health system capacity**	The overall capacity of the health system, including infrastructure, workforce, and resources, affects the ability to manage TB cases efficiently and effectively. A robust health system can better support TB control efforts.
**TB-specific health system capacity**	This includes the capacity to manage TB-specific needs, such as laboratories, TB clinics, and specialized health workers. It is critical for ensuring that TB control programs can operate effectively and adapt to new challenges.
**Health workforce**	A well-trained and adequately staffed health workforce is essential for providing quality TB care. Health workers need to be skilled in TB diagnosis, treatment, and patient management to ensure successful outcomes
**Governance**	Strong governance structures ensure that TB control programs are well-coordinated, policies are effectively implemented, and resources are appropriately allocated. Good governance also promotes accountability and transparency in TB control efforts.
**Access to medicine**	Reliable access to quality-assured TB medicines is crucial for successful treatment and prevention of drug resistance. Ensuring a steady supply of essential medicines is fundamental to TB control.
**Health information system**	Robust health information systems enable accurate tracking of TB cases, monitoring of treatment outcomes, and evaluation of program effectiveness. They support data-driven decision-making and help identify areas needing improvement
**Average temperature**	Temperature can influence TB transmission, as the bacteria thrive in certain climatic conditions. It can also affect human behavior and health, indirectly impacting TB incidence and outcomes
**Rainfall**	Rainfall patterns can affect living conditions and population movement, which in turn can influence TB transmission dynamics. For example, high rainfall can lead to damp living conditions that promote TB spread.
**Particulate matter**	Air pollution, particularly particulate matter, can impair lung function and increase susceptibility to respiratory infections, including TB. High levels of air pollution can exacerbate TB symptoms and complicate treatment.

Table S4: Summary of environmental factors and principal component analysis (PCA) results

Environmental characteristics

The average annual temperature of countries was found to be 20.2 ^{0}C ± 7.8, and the median (interquartile range) of annual rainfall was 1037mm (572, 1717.7mm). In addition, the median annual concentration of fine suspended particles in the air which is a measure of air pollution was 17.6 **μg/m^3.**

Table 1: Numeric summary of environmental factors

**Environmental factor s**	**Mean**	**SD**	**Min**	**P25**	**Median**	**P75**	**Max**
Average temperature(^{0}C)	20.2	7.8	-4.1	12.5	23.7	26.6	29.5
Rainfall(mm)	1209.3	870.8	22.8	572.0	1037.2	1717.7	5066.9
Particulate matter **(μg/m^3)**	22.1	14.2	5.3	10.9	17.6	28.7	65.1

Dimension reduction using PCA.

After assessing based on relevance and level of missing data, 33 variables were included as proxies of different health system building blocks. Then, a total of 14 principal factors that that explain more than 50% of the variation in each building block were generated and used to estimate the health system scores (Table 2). Results of the PCA are detailed in Additional file 3.

Table 2: Summary of the number of principal factors included and the amount of variance they explain.

Building block	Number of dimensions included	Number of Principal components retained	Amount of variation explained
TB Financing	6	3	67.75%
Routine health service delivery	4	1	62.46%
TB related health service delivery	3	2	69.77%
General health system capacity	4	2	55.00%
TB related health system capacity	2	1	69.75%
Health work force	2	1	83.75%
Governance	7	1	75.93%
Access to medicine	2	1	54.37%
Health information system	3	2	72.85%

TB: Tuberculosis

Table 3: Summary table of eigenvalues and proportion of variance for each principal factor in each building block of the health system

Principal factor	Eigenvalues	Variance percent	Cumulative variance percent
**TB related health expenditure**			
1	1.80	30.01	30.01
2	1.14	19.05	49.06
3	1.12	18.69	67.75
4	0.87	14.44	82.19
5	0.85	14.17	96.36
6	0.22	3.64	100
**Routine health service delivery**			
1	2.49	62.46	62.46
2	0.86	21.38	83.84
3	0.42	10.52	94.36
4	0.23	5.64	100
**TB related health service delivery**			
1	1.09	36.50	43.29
2	1.00	33.27	69.77
3	0.91	30.23	100
**General health system capacity**			
1	1.15	28.79	28.79
2	1.04	26.21	55.00
3	0.94	23.58	78.58
4	0.86	21.42	100
**TB related health system capacity**			
1	1.40	69.75	69.75
2	0.60	30.25	100
**Health workforce**			
1	1.67	83.75	83.75
2	0.30	14.98	100
**Governance**			
1	5.31	75.93	75.93
2	0.90	12.87	88.80
3	0.31	4.47	93.27
4	0.27	3.88	97.15
5	0.11	1.57	98.72
6	0.05	0.74	99.46
7	0.04	0.54	100

**Access to medicine**			
1	1.08	54.37	54.37
2	0.91	45.63	100
**Health information system**			
1	1.25	41.65	41.65
2	0.94	31.20	72.85
3	0.81	27.15	100

TB: Tuberculosis

Variables contribution for each principal factor

Table 3 shows the loadings(contribution) of each variable for each principal component and the communalities which is interpreted as the proportion of variation in that variable explained by the selected principal components mentioned in table 1. From the general health expenditure indicator variables, 97% of the variation in the domestic private health expenditure is explained by the first two principal factors. On the other hand, 88% of the variation for the variable average cost of drugs budgeted per patient for MDR-TB treatment is explained by the first three principal factors. From the governance indicator variables 96% of the variation is explained by the first principal factor. Similarly, the first principal factor explains 90% of the variation in TB treatment success rate which is the indicator of TB related health service delivery.

Table 3: The contribution of each variable for each principal factor and communality value.

Variable	PC 1	PC 2	PC 3	PC 4	PC 5	Communality
**TB related health expenditure**						
Average cost of drugs budgeted per patient for DSTB treatment	0.70	0.06	0.04	0.06	0.01	0.71
Average cost of drugs budgeted per patient for MDR-TB treatment	0.70	0.11	0.02	0.08	0.005	-0.71
Average cost of drugs budgeted per patient for TB preventive treatment	0.16	0.29	0.72	0.50	0.36	0.63
Average cost of drugs budgeted per patient for pre-XDR/XDR-TB	-0.10	0.55	0.51	-0.48	-0.45	0.57
Budget gap for TB)	-0.03	0.60	-0.42	0.55	-0.39	0.54
Total actual expenditure for TB	-0.13	0.66	-0.35	-0.31	0.58	0.58
**Governance**						
Logistic performance score	0.57	0.78	0.10	0.23	-0.03	0.33
Control of corruption index	0.96	-0.06	-0.09	-0.07	-0.25	0.92
Rule of low index	0.98	-0.03	-0.07	-0.10	-0.004	0.96
Government effectiveness index	0.96	0.12	-0.16	-0.10	0.05	0.92
Political stability index	0.81	-0.43	-0.17	0.36	0.06	0.66
Regulatory quality index	0.95	0.13	-0.01	0.11	0.20	0.90
Voice and accountability index	0.83	-0.29	0.48	0.01	0.00	0.69
**General health system capacity**						
Hospital per 100,000 population	0.64	-0.14	0.45	0.60		0.71
Health centre per 100,000 population	0.69	0.08	-0.006	-0.72		0.57
Health post per 100,000 population	0.25	0.71	-0.57	0.32		0.76
Hospital bed per 10,000 population	-0.21	0.68	-0.69	-0.13		0.41
**Routine health service delivery**						
Measles immunization coverage	0.57	-0.18	-0.32	-0.74		0.85
ART coverage	0.30	0.95	-0.07	0.03		0.25
DPT3 immunization coverage	0.56	-0.23	-0.43	0.67		0.81
Universal health coverage index	0.53	-0.10	0.84	0.07		0.72
**TB related health service delivery**						
TB case detection rate	0.68	-0.21	0.69			0.57
TB treatment success rate	0.18	0.98	0.12			0.90

BCG immunization coverage	0.70	-0.04	-0.71			0.77
**Health information system**						
Death cause registration	0.65	-0.11	0.75			0.72
Completeness of death cause registration	0.49	0.82	-0.31			0.30
Electronic health recording system in the country	-0.58	0.57	0.58			0.80
**TB related health system capacity**						
Number of TB basic management units	0.71	0.71				0.86
Number of TB diagnostic sites	0.71	-0.71				0.86
**Health workforce**						
Physicians per 1000 population	0.71	0.71				0.85
Nurses or midwifes per 1000 population	0.71	-0.71				0.85

ANC: Antenatal Care, ART: Anti-retroviral Treatment, ARI: Acute Respiratory Infection, BCG: Bacillus Calmette–Guérin, DSTB: Drug Susceptible Tuberculosis, MDRTB: Multi-Drug Resistant Tuberculosis, TB: Tuberculosis, XDR-TB: Extremely Drug Resistant Tuberculosis

Table S5: Health System Scores and percent reduction in TB incidence and mortality between 2015 and 2020 categorized by burden of TB at baseline (2015).

Health system scores (HSS) are generated as decile dimensions for 9 principal components (TB financing, routine health service delivery (HSD), TB specific HSD services, general health system capacity (HSC), TB specific HSC, access to medicines, workforce, governance and health information systems(HIS)), so scores can range from 0 (weakest health system on all 9 components) to 90 (strongest health system on all 9 dimensions).

#	Country Code	Country	% Reduction in TB incidence	% Reduction in TB mortality	TB financing	Routine HSD	TB HSD	HSC (General)	HSC (TB)	Work force	Access to medicine	Govern ance	HIS	**Total HSS**
**High-Burden Countries**														
1	NAM	Namibia	28.01	23.97	9	6	7	6	2	1	10	7	1	**49**
2	LSO	Lesotho	24.10	30.13	6	4	3	7	5	1	5	4	2	**37**
3	MMR	Myanmar	21.23	35.29	8	4	7	4	8	3	7	2	2	**45**
4	COG	Congo	0	11.21	4	2	3	4	2	4	3	1	6	**29**
5	LBR	Liberia	-1.95	18.89	1	1	3	4	4	1	9	3	2	**28**
6	NGA	Nigeria	0	11.76	10	1	2	9	10	1	2	2	2	**39**
7	KEN	Kenya	31.84	50.00	5	5	5	7	10	3	8	6	2	**51**
8	BGD	Bangladesh	1.36	33.31	7	4	10	3	9	3	10	3	1	**50**
9	THA	Thailand	7.98	22.73	8	10	7	8	9	4	10	6	10	**72**
10	KHM	Cambodia	25.14	8.33	4	6	10	6	6	3	5	3	4	**47**
11	COD	Democratic Republic of Congo	1.54	35.63	7	1	5	4	9	1	2	1	2	**32**
12	RUS	Russian Federation	31.34	43.64	10	9	7	10	10	9	7	3	8	**73**
13	AGO	Angola	4.37	27.59	8	1	1	6	7	4	7	2	2	**38**
14	ZMB	Zambia	18.41	29.57	8	5	6	6	7	3	9	4	2	**50**
15	BRA	Brazil	-4.65	-2.78	7	7	4	8	10	8	5	5	8	**62**
16	IND	India	13.36	5.56	8	4	4	6	10	4	1	6	1	**44**
17	MOZ	Mozambique	-1.94	59.60	5	3	9	4	8	2	8	2	2	**43**
18	ETH	Ethiopia	31.25	33.71	6	1	4	3	10	2	3	2	2	**33**
19	PAK	Pakistan	4.07	8.70	8	2	6	3	10	4	2	2	2	**39**
20	CHN	China	9.23	26.67	10	10	10	7	10	5	6	5	10	**73**
21	PNG	Papua New Guinea	3.24	10.34	9	1	2	8	7	4	4	4	2	**41**
22	SLE	Sierra Leone	2.93	46.58	1	2	5	7	6	2	10	3	2	**38**
23	TZA	United Republic of Tanzania	27.45	58.72	9	5	6	8	8	4	7	4	2	**53**
24	ZAF	South Africa	43.93	11.21	4	5	4	3	5	4	6	7	10	**48**
25	IDN	Indonesia	7.38	10.00	10	3	4	5	10	3	1	6	8	**50**

26	ZWE	Zimbabwe	20.25	2.33	5	5	6	7	8	3	2	1	2	**39**
27	PHL	Philippines	2.00	-7.41	9	2	3	5	10	6	6	5	2	**48**
28	CAF	Central African Republic	0	21.39	2	1	2	1	4	1	4	1	2	**18**
29	VNM	Viet Nam	11.56	33.33	6	8	8	9	9	6	8	5	5	**64**
**Low burden countries**														
1	OMN	Oman	20.45	6.98	5	3	6	4	4	6	9	7	7	**51**
2	ISR	Israel	47.50	-22.73	5	7	10	2	9	9	6	9	8	**65**
3	ALB	Albania	11.76	-5.41	2	6	9	1	1	7	2	6	2	**36**
4	QAT	Qatar	-41.67	6.45	5	7	7	8	5	8	7	8	8	**63**
5	MLI	Mali	8.77	24.55	8	1	4	6	4	1	4	2	2	**32**
6	PLW	Palau	29.67	29.33	1	2	4	1	2	5	6	7	6	**34**
7	UGA	Uganda	2.97	39.66	3	4	5	5	9	2	2	4	2	**36**
8	KNA	Saint Kitts & Nevis	8.51	5.00	8	8	1	10	1	9	1	8	7	**53**
9	RWA	Rwanda	4.92	10.84	4	7	9	7	8	2	5	6	2	**50**
10	EGY	Egypt	26.67	-27.66	2	5	7	2	6	4	5	3	10	**44**
11	URY	Uruguay	-6.67	22.86	6	9	8	7	5	7	8	9	10	**69**
12	CMR	Cameroon	17.92	-5.00	2	2	4	4	7	2	3	2	2	**28**
13	NOR	Norway	50.79	-44.00	4	10	9	10	3	10	3	10	9	**68**
14	BGR	Bulgaria	32.14	17.65	7	6	8	2	2	8	8	7	8	**56**
15	SWZ	Eswatini	50.77	59.29	9	5	6	3	4	5	10	4	1	**47**
16	SWE	Sweden	60.87	20.69	9	10	1	4	7	10	6	10	10	**67**
17	KAZ	Kazakhstan	14.81	9.30	9	7	10	8	6	7	9	5	6	**67**
18	IRN	Iran	18.75	8.33	9	7	8	5	8	5	9	2	8	**61**
19	AUT	Austria	34.67	38.46	1	9	3	2	7	9	8	10	7	**56**
20	MYS	Malaysia	-2.22	8.93	9	8	8	3	8	6	6	8	6	**62**
21	GEO	Georgia	29.29	-10.64	8	7	3	5	3	9	7	7	10	**59**
22	IRL	Ireland	27.40	2.44	9	8	1	4	10	10	8	10	5	**65**
23	MHL	Marshall Island	-61.00	-190.91	10	4	3	10	1	2	1	4	1	**36**
24	HRV	Croatia	49.23	-36.36	1	8	1	1	3	8	3	8	8	**41**
25	LUX	Luxembourg	3.28	-52.17	3	10	1	2	1	10	3	10	9	**49**
26	GIN	Guinea	-1.13	32.50	5	1	4	5	4	1	5	2	2	**29**
27	NIC	Nicaragua	16.00	14.29	8	7	9	5	8	4	10	2	2	**55**
28	LBN	Lebanon	-8.33	-45.83	2	3	9	4	1	5	10	2	5	**41**
29	CIV	Cote d'Ivoire	15.09	16.67	4	2	4	8	7	1	3	4	2	**35**
30	ESP	Spain	26.26	4.55	7	10	1	10	9	9	7	9	8	**70**
31	CZE	Czechia	29.09	59.26	6	8	6	9	2	9	10	9	6	**65**

32	DOM	Dominican Republic	25.45	32.84	10	4	6	10	9	6	4	6	1	**56**
33	VCT	Saint Vincent & the Grenadines	9.46	-8.33	1	3	2	5	1	8	7	9	7	**43**
34	JOR	Jordan	17.54	0	10	4	5	9	3	8	4	6	1	**50**
35	AND	Andorra	49.15	50.00	10	10	10	10	6	8	1	9	7	**71**
36	SOM	Somalia	5.47	14.10	4	1	1	10	5	1	1	1	2	**26**
37	LBY	Libya	-47.50	-18.18	8	4	2	6	1	5	9	1	10	**46**
38	GHA	Ghana	10.63	15.79	3	3	1	8	7	3	7	6	1	**39**
39	ITA	Italy	36.36	9.67	8	7	1	7	3	9	9	8	8	**60**
40	GMB	Gambia	9.25	13.33	1	3	7	6	3	2	9	3	2	**36**
41	DZA	Algeria	21.33	7.23	6	8	10	10	7	3	4	3	8	**59**
42	ROU	Romania	21.95	7.02	7	6	8	1	6	8	2	7	9	**54**
43	BTN	Bhutan	-1.23	-22.22	1	5	10	2	3	3	1	8	7	**40**
44	SVK	Slovakia	50.77	0	5	7	10	1	5	8	3	8	10	**57**
45	BLR	Belarus	48.00	33.33	8	8	9	9	4	10	7	3	6	**64**
46	STP	Sao Tome & Principe	24.36	40.00	2	7	3	8	1	4	1	5	1	**32**
47	TUR	Turkiye	16.67	21.13	4	9	9	3	7	6	3	5	7	**53**
48	SLV	El Salvador	-14.58	-27.78	3	5	8	3	5	4	7	5	9	**49**
49	LCA	Saint Lucia	75.56	18.75	9	9	1	2	4	6	6	8	6	**51**
50	POL	Poland	49.47	6.25	6	8	1	1	6	7	8	9	10	**56**
51	SSD	South Sudan	-2.20	51.47	1	1	2	9	2	1	1	1	2	**20**
52	FRA	France	2.38	9.52	6	10	1	6	4	10	2	9	8	**56**
53	TLS	Timor-Leste	-2.00	-3.88	1	2	5	5	3	4	10	4	6	**40**
54	AFG	Afghanistan	-2.12	30.00	3	1	5	3	9	2	8	1	2	**34**
55	ISL	Iceland	-16.67	-1.61	2	10	10	7	1	10	10	10	10	**70**
56	MKD	North Macedonia	29.41	0	3	5	8	9	1	7	2	6	7	**48**
57	IRQ	Iraq	27.03	14.81	7	2	8	8	6	1	4	1	2	**39**
58	COL	Colombia	-19.35	-21.88	4	7	5	6	9	9	7	6	2	**55**
59	PER	Peru	4.92	9.38	9	6	6	9	10	5	4	6	1	**56**
60	CUB	Cuba	11.27	14.29	7	10	5	2	7	8	9	4	7	**59**
61	SUR	Suriname	9.38	0	7	1	2	4	1	5	7	6	7	**40**
62	MNE	Montenegro	-6.67	0	2	2	6	4	1	7	10	7	10	**49**
63	SYR	Syrian Arab Republic	5.00	0	6	1	5	6	8	2	10	1	2	**41**
64	YEM	Yemen	-2.08	18.68	1	1	3	8	7	2	1	1	2	**26**
65	UZW	Uzbekistan	16.46	51.22	3	8	8	4	7	6	9	2	5	**52**
66	COM	Comoros	0	-42.03	1	3	1	4	6	3	6	2	7	**33**
67	ARE	United Arab Emirates	-3.95	1.47	7	9	9	8	3	7	2	9	2	**56**

68	MAR	Morocco	3.92	-17.65	3	9	10	6	5	4	1	5	1	**44**
69	BWA	Botswana	33.71	-8.89	8	7	5	2	3	5	1	8	6	**45**
70	GRC	Greece	4.25	-15.56	9	9	3	2	7	9	7	7	10	**63**
71	KIR	Kiribati	23.97	-18.42	2	3	9	10	1	2	10	7	1	**45**
72	CPV	Cabo Verde	39.06	-1.72	3	8	10	6	2	4	4	8	5	**50**
73	SGP	Singapore	-2.22	48.00	10	10	9	5	9	8	5	10	1	**67**
74	SYC	Seychelles	-13.40	-27.27	4	6	7	5	2	8	1	7	4	**44**
75	BHS	Bahamas	52.11	31.2	8	6	4	4	1	7	8	8	5	**51**
76	DEU	Germany	31.25	18.60	4	8	2	1	5	10	10	10	9	**59**
77	PAN	Panama	38.46	9.46	9	4	9	7	2	5	7	7	8	**58**
78	GNB	Guinea-Bissau	0	-4.62	2	2	2	10	5	2	5	2	2	**32**
79	TON	Tonga	37.5	15.38	8	1	4	9	4	9	2	7	1	**45**
80	UKR	Ukraine	19.78	11.76	7	2	3	6	8	8	9	3	4	**50**
81	SDN	Sudan	28.43	47.06	2	3	5	6	8	3	3	1	2	**33**
82	MWI	Malawi	28.43	47.06	5	5	5	4	6	2	10	4	2	**43**
83	ARM	Armenia	50.00	44.12	2	7	7	6	3	7	6	5	6	**49**
84	GUY	Guyana	15.05	0	8	9	5	9	2	1	5	5	5	**49**
85	BEL	Belgium	18.95	43.33	6	10	7	5	3	10	8	10	10	**69**
86	SEN	Senegal	4.88	0	3	4	8	3	5	2	2	6	2	**35**
87	SRB	Serbia	38.10	-10.00	7	6	8	1	2	8	1	6	9	**48**
88	CYP	Cyprus	8.06	-83.33	5	9	2	9	1	7	1	9	5	**48**
89	AZE	Azerbaijan	18.31	-24.59	2	5	7	10	4	9	1	2	9	**49**
90	NER	Niger	12.63	23.81	1	3	5	6	6	1	2	3	2	**29**
91	VEN	Venezuela	-46.67	-16.13	3	4	6	6	7	6	1	1	5	**39**
92	EST	Estonia	44.44	30.00	10	8	8	10	9	9	5	9	7	**75**
93	WSM	Samoa	44.55	47.31	5	2	10	7	6	7	9	8	1	**55**
94	PRT	Portugal	30.43	3.70	5	9	6	1	2	9	4	9	10	**55**
95	JPN	Japan	25.00	-16.67	6	9	4	1	10	9	9	10	10	**68**
96	DMA	Dominica	-45.45	0	10	6	1	9	8	6	6	8	8	**62**
97	KWT	Kuwait	13.64	-10.64	4	7	10	10	10	8	4	6	2	**61**
98	SLB	Solomon Islands	24.42	15.79	2	3	8	5	3	3	2	5	2	**33**
99	FJI	Fiji	-26.92	-68.29	2	6	4	9	1	5	6	7	9	**49**
10	TJK	Tajikistan	2.33	-4.17	5	7	9	9	5	5	1	2	5	**48**
101	GAB	Gabon	1.86	-12.24	5	3	1	7	1	4	4	3	8	**35**
102	KGZ	Kyrgyzstan	22.79	31.76	4	6	8	5	4	5	5	3	5	**45**
103	LKA	Sri Lanka	1.54	-2.86	3	7	7	10	5	5	5	5	9	**56**
104	TKM	Turkmenistan	4.08	-7.69	4	9	9	8	2	7	8	1	9	**57**

105	NLD	Netherlands	29.31	24.00	7	10	4	2	10	10	8	10	9	**70**
106	CHL	Chile	17.65	27.59	10	9	6	3	7	10	4	9	5	**63**
107	ATG	Antigua & Barbuda	45.24	0	3	9	1	2	3	7	3	8	5	**41**
108	MUS	Mauritius	7.69	-31.25	1	5	8	3	10	6	3	8	5	**49**
109	AUS	Australia	-21.67	4.17	5	10	9	8	8	10	5	10	7	**72**
110	GRD	Grenada	46.15	24	7	4	3	5	3	6	9	7	6	**50**
111	LTU	Lithuania	47.27	33.33	9	5	10	9	8	10	5	9	6	**71**
112	HND	Honduras	25.00	-21.74	7	5	7	4	9	3	9	3	1	**48**
113	DJI	Djibouti	38.96	37.21	5	2	6	5	4	2	10	2	2	**38**
114	ERI	Eritrea	20.59	15.00	4	6	8	3	4	3	7	1	2	**38**
115	PRY	Paraguay	-20.00	-19.05	3	4	4	7	6	6	1	4	9	**44**
116	HUN	Hungary	54.00	40.91	8	9	6	1	4	9	8	8	6	**59**
117	HTI	Haiti	13.40	-17.65	5	2	4	7	6	1	8	1	2	**36**
118	NRU	Nauru	-62.16	-108.79	2	5	10	10	5	6	2	4	8	**52**
119	MDV	Maldives	11.90	-31.25	3	8	5	10	8	8	10	5	5	**62**
120	BIH	Bosnia & Herzegovina	35.00	2.78	4	3	2	3	2	7	6	4	8	**39**
121	MNG	Mongolia	-2.10	9.09	9	6	4	8	2	7	5	6	7	**54**
122	BDI	Burundi	15.57	10.71	1	5	7	5	6	1	8	1	2	**36**
123	BRN	Brunei Darussalam	-40.68	-15.22	6	10	8	3	8	7	9	8	7	**66**
124	CHE	Switzerland	36.49	7.69	9	10	2	4	2	10	3	10	8	**58**
125	GBR	United Kingdom of Great Britain and Northern Ireland	31.00	15.25	10	8	7	6	5	10	6	10	7	**69**
126	MLT	Malta	-282.72	50.72	10	8	3	1	8	9	6	9	7	**61**
127	CRI	Costa Rica	9.09	-4.55	8	8	2	3	10	5	2	8	9	**55**
128	FSM	Micronesia (Federated States of)	36.97	36.92	7	3	7	9	2	4	5	7	6	**50**
129	MEX	Mexico	-9.09	-20.83	3	6	3	9	9	8	9	5	6	**58**
130	MDA	Republic of Moldova	27.45	5.38	3	4	7	7	4	7	6	5	2	**45**
131	VUT	Vanuatu	39.68	30.43	1	3	6	10	1	3	2	5	1	**32**
132	ECU	Ecuador	-23.08	-14.89	6	4	5	2	9	6	7	4	7	**50**
133	NZL	New Zealand	1.39	42.55	6	9	10	8	9	10	4	10	7	**73**
134	KOR	Republic of Korea	37.97	28.30	7	10	10	10	7	7	4	9	9	**73**
135	MRT	Mauritania	18.69	36.00	5	1	3	1	3	2	3	2	2	**22**
136	SAU	Saudi Arabia	32.50	22.22	9	7	9	2	2	6	9	6	1	**51**
137	TCD	Chad	0	11.43	4	1	2	3	5	1	4	1	2	**23**
138	LVA	Latvia	42.50	39.34	7	6	1	2	3	8	6	9	6	**48**

139	CAN	Canada	-1.89	34.21	10	8	9	5	1	9	9	10	10	**71**
140	TUN	Tunisia	5.26	-21.43	7	6	9	8	3	5	10	6	2	**56**
141	ARG	Argentina	-19.23	-16.67	10	5	2	1	9	8	2	5	10	**52**
142	SVN	Slovenia	43.06	-27.78	3	9	7	2	2	10	4	9	9	**55**
143	BOL	Bolivia	10.26	14.29	6	3	5	7	9	4	3	3	5	**45**
144	NPL	Nepal	12.31	15.15	2	4	5	1	8	4	3	4	1	**32**
145	GNQ	Equatorial Guinea	-2.19	9.43	1	1	1	2	4	2	5	1	1	**18**
146	FIN	Finland	35.71	9.68	10	10	2	5	7	10	7	10	5	**66**
147	COK	Cook Islands	0	-36.00	6	3	2	1	6	9	8	10	6	**51**
148	LAO	Lao People's Democratic Republic	18.13	50	1	3	6	9	6	3	7	3	6	**44**
149	JAM	Jamaica	46.67	34.04	5	6	3	10	3	3	2	7	5	**44**
150	DNK	Denmark	24.62	41.18	6	10	2	7	4	10	8	10	9	**66**
151	NIU	Niue	-65.50	33.33	6	2	2	2	6	6	4	10	6	**44**
152	BRB	Barbados	-100.00	0	10	7	3	1	10	6	9	8	10	**64**
153	TUV	Tuvalu	-43.00	-11.76	3	5	10	2	9	5	6	7	9	**56**
154	GTM	Guatemala	-3.85	-14.81	4	3	6	3	8	3	8	3	9	**47**
155	BEN	Benin	8.33	20.00	4	2	7	7	4	1	4	5	2	**36**
156	BHR	Bahrain	13.33	-33.96	1	9	1	7	5	5	5	6	8	**47**
157	BLZ	Belize	8.00	3.33	2	5	4	8	6	5	3	4	5	**42**
158	BFA	Burkina Faso	11.54	0	10	4	6	8	5	1	6	4	2	**46**
159	MDG	Madagascar	-2.15	15.09	2	1	3	4	7	2	1	3	2	**25**
160	TTO	Trinidad & Tobago	-12.50	-5.26	6	8	3	1	1	6	5	7	4	**41**
161	USA	United States of America	25.00	0	9	6	9	9	10	7	7	9	8	**74**
162	TGO	Togo	30.77	57.29	1	2	8	3	5	1	2	3	2	**27**

Table S6: Univariable and adjusted effect of health system and environmental factors on percent reduction of TB incidence and mortality

Table 1: Univariable and adjusted effect of health system and environmental factors on percent reduction of TB incidence and achievement of WHO end TB target, ≥20% reduction in TB incidence between 2015 and 2020.

**Variables**	**TB Incidence (Outcome 1)**			
	**% Reduction in TB incidence**		**Achieve ≥20% reduction(yes/no)**	
	Unadjusted β (95% CI)	*Adjusted β (95% CI)*	*Unadjusted OR (95% CI)*	*Adjusted OR (95% CI)*
**General Health expenditure**				

Domestic general government health expenditure (% of current health expenditure)	0.073(-0.135, 0.280)	0.004(-0.307, 0.314)	1.020(1.006, 1.035) **	1.005(0.985, 1.025)
Out-of-pocket expenditure per capita (current US$)	-0.0008(-0.017, 0.015)	-0.009(-0.038, 0.019)	1.002(1.0003, 1.003) *	1.0004(0.999, 1.002)
Current health expenditure (% of GDP)	-0.256(-1.845, 1.334)	-0.466(-2.132, 1.199)	1.151(1.038, 1.288) *	1.106(0.995, 1.237)
External health expenditure (% of current health expenditure)	-0.065(-0.304, 0.174)	0.102(-0.222, 0.426)	0.998(0.977, 1.019)	1.036(1.006, 1.069) *
Domestic private health expenditure (% of current health expenditure)	-0.080(-0.330, 0.171)	-0.056(-0.320, 0.209)	0.977(0.960, 0.993) **	0.982(0.964, 0.999) *
Out-of-pocket expenditure (% of current health expenditure)	-0.104(-0.351, 0.142)	-0.082(-0.346, 0.183)	0.976(0.959, 0.991) **	0.982(0.964, 0.999) *
Current health expenditure per capita (current US$)	0.001(-0.003, 0.004)	9.1e-04(-0.003, 0.005)	1.0003(1.0001, 1.0005) **	1.0001(0.999, 1.0004)
**TB related financing**				
Average cost of drugs budgeted per patient for DSTB treatment	-0.032(-0.057, -0.007) **	-0.033(-0.057, -0.009)	0.998(0.993, 1.001)	0.998(0.991, 1.001)
Average cost of drugs budgeted per patient for MDR-TB treatment	-0.0002(-0.0004, -0.0001) ***	-2.3e-04(-0.0004, -0.0001) ***	0.999 (NA, 1.000)	0.999 (NA, 1.000)
Average cost of drugs budgeted per patient for pre-XDR/XDR-TB	0.0003(-0.0001, 0.0007)	2.1e-04(-2.2e-04, 7.0e-04)	1.0001(0.999, 1.0006)	1.0003(0.9999, 1.0005)
Average cost of drugs budgeted per patient for TB preventive treatment	0.070(-0.035, 0.175)	0.070(-0.039, 0.178)	1.011(0.998, 1.029)	1.009(0.997, 1.028) 1
Budget gap for TB	-2.2e-08(-1.3e-07, 8.4e-08)	-3.1e-08(-1.5e-07, 8.5e-07)	1(1, 1)	1(0.999, 1.000)
Total actual expenditure for TB	9.5e-09(-1.4e-08, 3.3e-08)	8.4e-09(-1.7e-08, 3.3e-08)	1(1, 1)	1(1, 1)
**Routine health service delivery**				
Measles immunization coverage	0.146(-0.173, 0.465)	-0.09(-0.288, 0.470)	1.024(1.003, 1.049) *	1.005(0.980, 1.032)
ART coverage	0.088(-0.099, 0.277)	0.081(-0.113, 0.274)	1.021(1.003, 1.041) *	1.017(0.998, 1.037)
DPT3 immunization coverage	0.124(-0.204, 0.452)	-0.057(-0.568, 0.453)	1.036(1.011, 1.064) **	1.019(0.992, 1.049)
Universal health coverage index	0.027(-0.367, 0.421)	-0.378(-1.419, 0.663)	1.031(1.011, 1.053) **	0.989(0.939, 1.040)
**TB related health service delivery**				
TB case detection rate	0.158(-0.115, 0.430)	0.130(-0.204, 0.464)	1.037(1.017, 1.058) ***	1.025(1.002, 1.051) *
TB treatment success rate	-0.070(-0.252, 0.111)	0.016(-0.176, 0.208)	0.992(0.976, 1.008)	1.003(0.986, 1.022)
BCG immunization coverage	-0.179(-0.561, 0.203)	-0.209(-0.599, 0.180)	1.003(0.981, 1.027)	0.998(0.975, 1.022)
**Health system capacity and workforce**				
Hospital per 100000 population	-0.585(-2.249, 1.079)	-0.549(-2.237, 1.138)	0.912(0.729, 1.027)	0.842(0.652, 1.018)
Health centre per 100000 population	-0.033(-0.530, 0.595)	-0.004(-0.525, 0.614)	1.005(0.983, 1.030)	1.008(0.985, 1.033)
Health post per 100000 population	0.049(-0.139, 0.237)	0.058(-0.134, 0.251)	1.003(0.993, 1.013)	1.002(0.991, 1.013)

Hospital bed per 10000 population	0.257(-0.100, 0.614)	0.311(-0.115, 0.737)	**1.043(1.021, 1.068) *****	**1.038(1.013, 1.067) ****
Number of TB basic management units	-0.001(-0.006, 0.003)	-0.001(-0.007, 0.003)	0.999(0.998, 1.0002)	0.999(0.998, 1.0001)
Number of TB diagnostic sites	0.0003(-0.004, 0.004)	-0.0004(-0.004, 0.003)	0.999(0.998, 1.00005)	0.999(0.998, 1.00005)
Physician per 1000 population	0.127(-0.128, 0.382)	0.212(-0.246, 0.671)	**1.029(1.015, 1.045) *****	1.018(0.994, 1.043)
Nurse or midwife per 1000 population	-0.050(-1.953, 1.852	0.073(-1.938, 2.085)	**1.123(1.033, 1.232) ****	1.023(0.906, 1.165)
**Governance**				
Logistic performance score	5.480(-0.048, 11.007)	5.913(-0.634, 12.460)	**1.550(1.065, 2.334) ***	1.084(0.708, 1.698)
Control of corruption index	2.995(-1.705, 7.694)	3.112(-3.664, 9.888)	**1.788(1.311, 2.484) *****	1.471(0.945, 2.316)
Rule of low index	3.780(-1.002, 8.561)	5.101(-2.302, 12.504)	**1.973(1.425, 2.793) *****	**1.781(1.084, 3.000) ***
Government effectiveness index	3.915(-0.803, 8.634)	7.104(-1.617, 15.826)	**1.781(1.300, 2.487) *****	1.404(0.788, 2.534)
Political stability index	1.432(-3.360, 6.223)	0.196(-6.003, 6.395)	**1.810(1.299, 2.589) *****	1.505(0.990, 2.347)
Regulatory quality index	3.752(-1.024, 8.528)	5.356(-2.403, 13.114)	**1.792(1.303, 2.512) *****	1.412(0.845, 2.432)
Voice and accountability index	2.408(-2.274, 7.090)	1.745(-4.036, 7.526)	**1.888(1.374, 2.646) *****	**1.599(1.091, 2.397) ***
Policy and action plan for CRD (no)	5.190(-5.045, 15.424)	6.252(-4.210, 16.714)	0.891(0.471, 1.671)	1.059(0.541, 2.077)
**Access to medicine**				
Children treated with oral rehydration therapy	0.116(-0.068, 0.301)	0.116(-0.076, 0.309)	1.013(0.988, 1.038)	1.010(0.984, 1.036)
Pregnant women treated for syphilis	0.061(-0.225, 0.347)	0.077(-0.212, 0.367)	1.008(0.992, 1.025)	1.006(0.991, 1.024)
**Health information system**				
Death cause registration	0.693(-0.975, 2.361)	0.656(-1.070, 2.383)	1.047(0.975, 1.134)	1.055(0.980, 1.150)
EHR in the country(no)	-4.230(-18.125, 9.665)	-3.504(-18.020, 11.011)	0.911(0.433, 1.914)	1.337(0.599, 3.037)
Completeness of death cause registration	0.173(-0.205, 0.550)	0.144(-0.276, 0.564)	**1.030(1.009, 1.063) ***	1.021(0.996, 1.052)
**Environmental factors**				
Particulate matter	-0.138(-0.426, 0.186)	-0.091(-0.447, 0.265)	**0.952(0.926, 0.976) *****	**0.963(0.936, 0.988) ****
Mean temperature	**-0.971(-1.546, -0.395) ****	**-1.179(-1.860, -0.499) *****	**0.824(0.623, 0.925) *****	**0.631(0.457, 0.769) ***
Rainfall(precipitation)	-0.005(-0.010, 0.0007)	-0.005(-0.009, 0.0008)	1.0003(0.999, 1.0006)	1.0003(0.999, 1.0007)

ANC: Antenatal Care, ART: Anti-retroviral Treatment, ARI: Acute Respiratory Infection, BCG: Bacillus Calmette–Guérin, CRD: Chronic Respiratory Disease, DSTB: Drug Susceptible Tuberculosis, EHR: Electronic health record, GDP: Gross Domestic Product, MDRTB: Multi-Drug Resistant Tuberculosis, TB: Tuberculosis, XDR-TB: Extremely Drug-Resistant Tuberculosis

**** p-value < 0.0001, **p-value <0.001, *p-value<0.05*

Note: univariable and adjusted analysis was conducted before data imputation to assess the individual impact of each factor and to select variables to be included in the principal component analysis.

Table 2: Univariable and adjusted effect of health system and environmental factors on percent reduction of TB related mortality and achievement of WHO end TB target, ≥35% reduction in TB related mortality between 2015 and 2020.

**Variables**	**TB mortality (Outcome 2)**			
	**% Reduction in TB mortality**		**Achieve ≥ 35% reduction(yes/no)**	
	Unadjusted β (95% CI)	*Adjusted β (95% CI)*	*Unadjusted OR (95% CI)*	*Adjusted OR (95% CI)*
**General Health expenditure**				
Domestic general government health expenditure (% of current health expenditure)	**-0.200(-0.386, -0.015) ***	-0.078(-0.350, 0.194)	0.984(0.967, 1.001)	0.990(0.964, 1.015)
Out-of-pocket expenditure per capita (current US$)	-0.068(-0.163, 0.027)	0.116(-0.067, 0.299)	0.996(0.989, 1.003)	1.005(0.991, 1.020)
Current health expenditure (% of GDP)	-1.294(-2.721, 0.133)	-0.0864(-2.326, 0.598)	0.963(0.832, 1.096)	0.995(0.862, 1.131)
External health expenditure (% of current health expenditure)	**0.347(0.056, 0.638) ***	0.203(-0.186, 0.592)	**1.044(1.019, 1.070) *****	**1.048(1.014, 1.085) ****
Domestic private health expenditure (% of current health expenditure)	0.190(-0.084, 0.464)	0.082(-0.201, 0.365)	0.998(0.978, 1.018)	0.991(0.970, 1.011)
Out-of-pocket expenditure (% of current health expenditure)	0.205(-0.064, 0.474)	0.120(-0.162, 0.403)	1.002(0.982, 1.021)	0.996(0.975, 1.017)
Current health expenditure per capita (current US$)	-0.001(-0.004, 0.002)	0.002(-0.002, 0.006)	0.999(0.998, 1.001)	1.001(0.999, 1.004) 1
**TB related financing**				
Average cost of drugs budgeted per patient for DSTB treatment	**-0.104(-0.137, -0.071) *****	**-0.096(-0.128, -0.064) *****	0.994(0.980, 1,001)	0.998(0.986, 1.002)
Average cost of drugs budgeted per patient for MDR-TB treatment	**-6.8e-04(-8.4e-04, -5.2e-04) *****	**-6.6e-04(-8.1e-04, 5.2e-04) *****	0.999(0.998, 1.001)	0.999(0.996, 1.001)
Average cost of drugs budgeted per patient for pre-XDR/XDR-TB	4.6e-04(-2.6e-04, 0.001)	6.3e-04(-6.5e-05, 0.001)	0.999(0.997, 1.003)	0.999(0.996, 1.0001)
Average cost of drugs budgeted per patient for TB preventive treatment	0.027(-0.155, 0.209)	0.091(-0.087, 0.268)	0.981(0.937, 1.005)	0.987(0.936, 1.012)
Budget gap for TB	6.0e-08(-1.1e-07, 2.3e-07)	-1.7e-08(-2.0e-07, 1.6e-07)	1(1, 1)	1(0.999, 1.001)
Total actual expenditure for TB	2.5e-08(-1.6e-08, 6.6e-08)	2.2e-08(-2.0e-08, 6.4e-08)	1(1, 1)	1(0.999, 1.001)
**Routine health service delivery**				
Measles immunization coverage	-0.343(-0.689, 0.004)	-0.193(-0.598, 0.211)	1.003(0.978, 1.033)	1.014(0.984, 1.048)
ART coverage	-0.022(-0.193, 0.236)	0.078(-0.125, 0.281)	1.010(0.988, 1.034)	1.014(0.990, 1.041)
DPT3 immunization coverage	-0.140(-0.435, 0.156)	0.055 (-0.283, 0.392)	1.004(0.979, 1.033)	1.023(0.991, 1.061)
Universal health coverage index	**-0.301(-0.566, -0.036) ***	-0.140(-0.829, 0.549)	0.978(0.954, 1.002)	0.981(0.920, 1.045)
**TB related health service delivery**				
TB case detection rate	0.010(-0.237, 0.256)	0.279 (-0.012, 0.569)	1.005(0.984, 1.029)	**1.032(1.001, 1.069) ***

TB treatment success rate	**0.394(0.167, 0.621) *** **	**0.326(0.087, 0.565) ** **	1.024(0.999, 1.057)	1.018(0.992, 1.051)
BCG immunization coverage	-0.112(-0.412, 0.188)	-0.035(-0.330, 0.260)	1.005(0.979, 1.038)	1.045(0.988, 1.067)
**Health system capacity and workforce**				
Hospital per 100000 population	-0.771(-1.938, 0.396)	-0.705(-1.871, 0.462)	0.853(0.580, 1.046)	0.904(0.611, 1.051)
Health centre per 100000 population	**-0.618(-0.939, -0.298) *** **	**-0.605(-0.923, -0.286) *** **	0.951(0.847, 1.007)	0.950(0.839, 1.008)
Health post per 100000 population	0.095(-0.058, 0.247)	0.119(-0.036, 0.273)	1.006(0.995, 1.018)	1.008(0.996, 1.019)
Hospital bed per 10000 population	0.139(-0.074, 0.352)	0.207(-0.044, 0.459)	1.017(0.995, 1.038)	1.016(0.991, 1.041)
Number of TB basic management units	0.002(-0.007, 0.011)	3.0e-04(-8.3e-03, 0.009)	1.001(0.999, 1.006)	0.999(0.998, 1.005)
Number of TB diagnostic sites	0.001(-0.002, 0.004)	-6.4e-04(-0.004, 0.003)	1.002(0.999, 1.005)	0.999(0.997. 1.002)
Physician per 1000 population	-0.061(-0.277, 0.156)	0.183(-0.200, 0.567)	1.0001(0.983, 1.017)	1.003(0.973, 1.034)
Nurse or midwife per 1000 population	-0.346(-1.687, 0.996)	0.964(-0.558, 2.485)	0.988(0.879, 1.090)	1.038(0.880, 1.202)
**Governance**				
Logistic performance score	3.566(-1.565, 8.698)	**8.089(2.302, 13.877) ** **	0.964(0.627, 1.528)	1.147(0.669, 2.228)
Control of corruption index	-3.127(-8.261, 3.760)	3.131(-4.077, 10.338)	0.954(0.645, 1.383)	1.581(0.896, 2.836)
Rule of low index	-0.576(-4.912, 2.472)	**7.432(1.004, 13.860) * **	1.050(0.706 1.547)	**2.350(1.189, 4.923) * **
Government effectiveness index	0.037 (-4.245, 4.319)	**12.704(5.234, 20.173) *** **	0.934(0.633, 1.362)	**2.585(1.195, 4.900) * **
Political stability index	-2.492(-6.802, 1.817)	1.903(-3.524, 7.331)	0.995(0.686, 1.469)	1.535(0.914, 2.699)
Regulatory quality index	-0.269(-4.600, 4.062)	**8.905(2.197, 15.613) ** **	0.880(0.592, 1.291)	1.503(0.765, 3.181)
Voice and accountability index	-3.352(-7.558, 0.854)	-0.620(-5.691, 4.452)	0.850(0.586, 1.231)	1.039(0.654, 1.696)
Policy and action plan for CRD (no)	-8.493(-17.493, 0.507)	-8.188(-17.247, 0.872)	0.711(0.299, 1.601)	0.740(0.304, 1.713)
**Access to medicine**				
Children treated with oral rehydration therapy	0.235(-0.016, 0.486)	0.216(-0.024, 0.456)	1.028(0.999 1.059)	**1.031(1.005, 1.068) * **
Pregnant women treated for syphilis	**0.262(0.054, 0.471) * **	**0.306(0.104, 0.507) ** **	1.003(0.983, 1.027)	1.007(0.985, 1.035)
**Health information system**				
Death cause registration	-0.616(-1.542, 0.311)	-0.746(-1.682, 0.190)	0.910(0.731, 1.045)	0.862(0.731, 1.045)
EHR in the country(no)	1.473(-8.496, 11.442)	1.034(-9.175, 11.244)	1.526(0.588, 4.171)	1.688(0.625, 4.801)
Completeness of death cause registration	0.176(-0.144, 0.496)	0.135(-0.218, 0.488)	1.063(1.007, 1.167)	1.038(0.987, 1.134)
**Environmental factors**				
Particulate matter	0.271(-0.082, 0.624)	0.137(-0.244, 0.517)	0.990(0.962, 1.016)	0.976(0.943, 1.006)
Mean temperature	-0.364(-0.891, 0.164)	**-0.905(-1.500, -0.308) ** **	0.991(0.947, 1.041)	0.958(0.903, 1.016)
Rainfall(precipitation)	-0.005(-0.009, 6.8e-05)	**-0.006(-0.010, -0.0009) * **	1.001(0.999, 1.004)	0.999(0.998, 1.003)

ANC: Antenatal Care, ART: Anti-retroviral Treatment, ARI: Acute Respiratory Infection, BCG: Bacillus Calmette–Guérin, CRD: Chronic Respiratory Disease, DSTB: Drug Susceptible Tuberculosis, EHR: Electronic health record, GDP: Gross Domestic Product, MDRTB: Multi-Drug Resistant Tuberculosis, TB: Tuberculosis, XDR-TB: Extremely Drug-Resistant Tuberculosis

*Significance levels: *** p-value < 0.0001, **p-value <0.001, *p-value<0.05*
Note: univariable and adjusted analysis was conducted before data imputation to assess the individual impact of each factor and to select variables to be included in the principal component analysis.

Table S7: Linier and logistic regression outputs on the effect of health system building blocks on TB control.

We used backward stepwise regression which is a variable selection technique that begins with a full model, which includes all potential predictor variables and iteratively removing the least significant variables one at a time based on a pre-specified criterion. In our analysis, we started with a comprehensive set of health system and environmental variables believed to influence TB incidence and mortality. We then applied the backward stepwise procedure using a significance level ($p < 0.10$) for variable retention and a p-value of 0.2 for variable exclusion. This means that in each step variables the variable with the highest p-value (above 0.2) was removed, and the model was re-fitted. This process continued until all remaining variables had p-values below the specified threshold, indicating their statistical significance in predicting the outcomes.

**linear regression output for the effect of all health system factors on percent reduction in TB incidence and mortality before applying backward stepwise regression**						
**Health system factors**	**Percent reduction in incidence**			**Percent reduction in mortality**		
	All	*Low burden*	*High burden*	*All*	*Low burden*	*High burden*
TB specific financing	-6.7(-13.7, 0.3)	-6.3(-14.2, 1.6)	-2.8(-22.1, 1.6)	-2.7(-8.5, 3.1)	-3.2(-9.6, 3.3)	14.4(-8.1, 36.9)
Routine health service delivery	1.9(-2.6, 6.4)	1.7(-3.7, 7.0)	2.6(-6.9, 12.1)	-2.3(-6.0, 1.4)	-3.3(-7.7, 1.1)	9.6(-1.5, 20.7)
TB health service delivery	0.8(-6.0, 7.6)	0.7(-6.9, 8.2)	-1.3(-18.4, 15.7)	**6.6(1.0, 12.1) ***	**6.4(0.2, 12.5) ***	-7.2(-27.1, 12.7)
General health system capacity	4.4(-4.4, 13.1)	4.2(-5.3, 13.7)	6.3(-37.4, 50.0)	-5.3(-12.4, 1.9)	-5.7(-13.5, 2.1)	-1.1(-6.2, 39.8)
TB specific health system capacity	-2.1(-6.4, 2.3)	-5.7(-13.4, 2.3)	0.2(-3.0, 3.4)	0.03(-3.5, 3.6)	0.8(-5.7, 7.3)	0.7(-3.1, 4.4)
Health work force	-1.0(-8.6, 6.7)	-0.4(-9.2, 8.4)	9.2(-24.1, 14.5)	1.0(-5.3, 7.2)	0.5(-2.2, 11.4)	5.3(-1.2, 22.7)
Governance	1.7(-1.8, 5.2)	1.6(-2.4, 5.6)	2.8(-5.2, 8.2)	**4.2(1.3, 7.1) ****	**4.6(0.5, 6.9) ***	0.06(-7.8, 7.9)
Access to medicine	0.4(-4.2, 5.0)	1.1(-4.2, 6.3)	-2.2(-9.1, 4.7)	**5.6(1.7, 9.3) ****	**5.2(0.9, 9.7) ***	**8.9(0.8, 16.9) ***
Health information system	-4.5(-9.5, 0.5)	-5.0(-10.9, 0.8)	-1.4(-7.5, 3.7)	-2.5(-6.6, 1.6)	-3.2(-8.0, 1.5)	3.1(-3.4, 9.7)
**Environmental factors**						
Average temperature	**-1.4 (-2.2, -0.5) ****	**-1.4(-2.4, -0.5) ****	-0.7(-1.9, 0.5)	**-0.7(-1.4, -0.5) ***	-0.7(-1.5, 0.05)	-0.5(-1.9, 0.9)
Rainfall	-0.002(-8.3, 0.04)	-0.09(-0.1, 0.007)	-0.004(-0.01, 0.007)	-0.005(-0.01, -0.0004)	-0.006(-0.01, 0.007)	0.001(-0.08, 0.01)
Particulate matter	-0.2(-5.6, 0.3)	-0.1(-0.6, 0.4)	-0.4(-0.8, 0.1)	-0.1(-0.5, 0.2)	-0.09(-0.5, 0.3)	-0.3(-0.8, 0.2)
R^2 of the model	0.13	0.13	0.28	0.25	0.21	0.41
**Logistic regression output for the effect of all health system factors on the achievement of milestone one of WHO end TB targets**						
**Health system factors**	**Achieve 20% reduction in incidence**			**Achieve 35% reduction in mortality**		
	All	*Low burden*	*High burden*	*All*	*Low burden*	*High burden*
TB specific financing	0.9(0.5, 1.5)	0.9(0.6, 1.7)	6.2(0.1, 11.1)	1.7(0.9, 3.1)	1.4(0.7, 2.7)	9.1(0.3, 27.5)
Routine health service delivery	1.1(0.8, 1.5)	0.9(0.7, 1.4)	**4.3(2.8, 6.63) ***	1.2(0.8, 1.8)	1.03(0.7, 1.7)	4.3(0.1, 34.6)

TB health service delivery	1.2(0.8, 1.9)	1.2(0.7, 1.9)	1.5(0.67, 3.0)	1.7(0.8, 3.7)	1.5(0.8, 3.4)	1.3(0.03, 28.4)
General health system capacity	0.9(0.4, 1.6)	0.9(0.4, 1.7)	1.3(0.25, 12.4)	0.5(0.1, 1.4)	0.5(0.1, 1.3)	1.4(0.7, 30.1)
TB specific health system capacity	0.7(0.4, 1.1)	0.6(0.3, 1.1)	0.5(0.15, 9.5)	0.9(0.6, 1.3)	0.7(0.2, 1.5)	1.2(0.6, 9.8)
Health work force	0.9(0.6, 1.5)	0.8(0.5, 1.5)	0.6(0.07, 7.9)	1.7(0.8, 3.2)	1.1(0.5, 2.3)	1.7(0.2, 43.6)
Governance	1.2 (0.9, 1.6)	**1.3(1.1, 1.7) ***	0.6(0.3, 5.1)	1.2(0.9, 1.7)	**1.4(1.1, 2.1) ***	1.1(0.02, 12.9)
Access to medicine	1.02(0.7, 1.4)	1.1(0.8, 1.6)	1.1(0.3, 7.1)	1.3(0.9, 2.0)	1.3(0.8, 2.1)	5.3(0.4, 23.1)
Health information system	0.7(0.5, 1.1)	0.8(0.5, 1.1)	0.4(0.04, 1.5)	0.7(4.4, 1.1)	0.7(0.4, 1.1)	0.5(0.1, 19.1)
**Environmental factors**						
Average temperature	**0.6(0.5, 0.8) ***	0.9(0.8, 1.4)	0.9(0.5, 1.2)	0.9(0.8, 1.1)	0.9(0.8, 1.1)	0.8(0.6, 18.1)
Rainfall	**0.8(0.4, 0.9) ***	0.9(0.6, 1.7)	0.9(0.7, 2.7)	0.9(0.7, 1.2)	0.9(0.6, 1.2)	0.4(0.02, 19.7)
Particulate matter	**0.7(0.6, 0.9) ****	**0.8(0.7, 0.9) ***	0.8(0.7, 9.1)	0.9(0.8, 1.3)	0.9(0.8, 1.4)	0.5(0.1, 10.4)
**Logistic regression output for the effect of selected health system factors on the achievement of milestone one of WHO end TB targets after applying back ward stepwise regression.**						
**Health system factors**	**Achieve 20% reduction in incidence**			**Achieve 35% reduction in mortality**		
	All	*Low burden*	*High burden*	*All*	*Low burden*	*High burden*
TB specific financing	Not selected	Not selected	Not selected	1.6(0.8, 3.1)	Not selected	Not selected
Routine health service delivery	Not selected	Not selected	**1.7(1.3, 4.1) ***	Not selected	Not selected	Not selected
TB health service delivery	Not selected	Not selected	0.5(0.3, 3.4)	**1.9(1.1, 4.0) ***	Not selected	Not selected
General health system capacity	Not selected	Not selected	Not selected	0.4(0.07, 1.1)	Not selected	Not selected
TB specific health system capacity	Not selected	0.7(0.4, 0.9)	0.6(0.2, 1.2)	Not selected	Not selected	Not selected
Health work force	0.6(0.3, 1.1)	Not selected	Not selected	Not selected	Not selected	Not selected
Governance	1.2(0.9, 1.6)	1.2(0.9, 1.6)	Not selected	Not selected	**1.4(1.1, 2.0) ***	Not selected
Access to medicine	Not selected	Not selected	0.2(0.01, 8.6)	Not selected	Not selected	Not selected
Health information system	0.7(0.4, 1.3)	0.73(0.5, 1.1)	Not selected	0.6(0.4, 1.1)	0.7(0.4, 1.1)	Not selected
**Environmental factors**						
Average temperature	**0.7(0.6, 0.9) ***	**0.8(0.7, 0.9) ***	Not selected	0.9(0.8, 1.1)	Not selected	Not selected
Rainfall	**0.8(0.3, 0.9) ****	Not selected	Not selected	Not selected	Not selected	Not selected
Particulate matter	**0.6(0.4, 0.8) ****	0.9(0.8, 1.1)	Not selected	0.9(0.8, 1.3)	Not selected	Not selected
AIC	243.3	203.4	34.6	172.3	136.4	

- *All:* model for all 191 countries and these models are adjusted for TB burden, total current health expenditure as percent of GDP and HDI of countries in 2015, *Low burden:* model for 162 countries classified as low TB burden by WHO in 2015, High burden: model for 29 countries classified as high TB burden by WHO in 2015. Models for both high and low burden countries are adjusted for total current health expenditure as percent of GDP and HDI of countries in 2015.
- All linier regression outputs report β coefficients, while all logistic regression outputs report adjusted odds ratio.
- Significance: * p-value <0.05 and ** <0.01

Additional figures

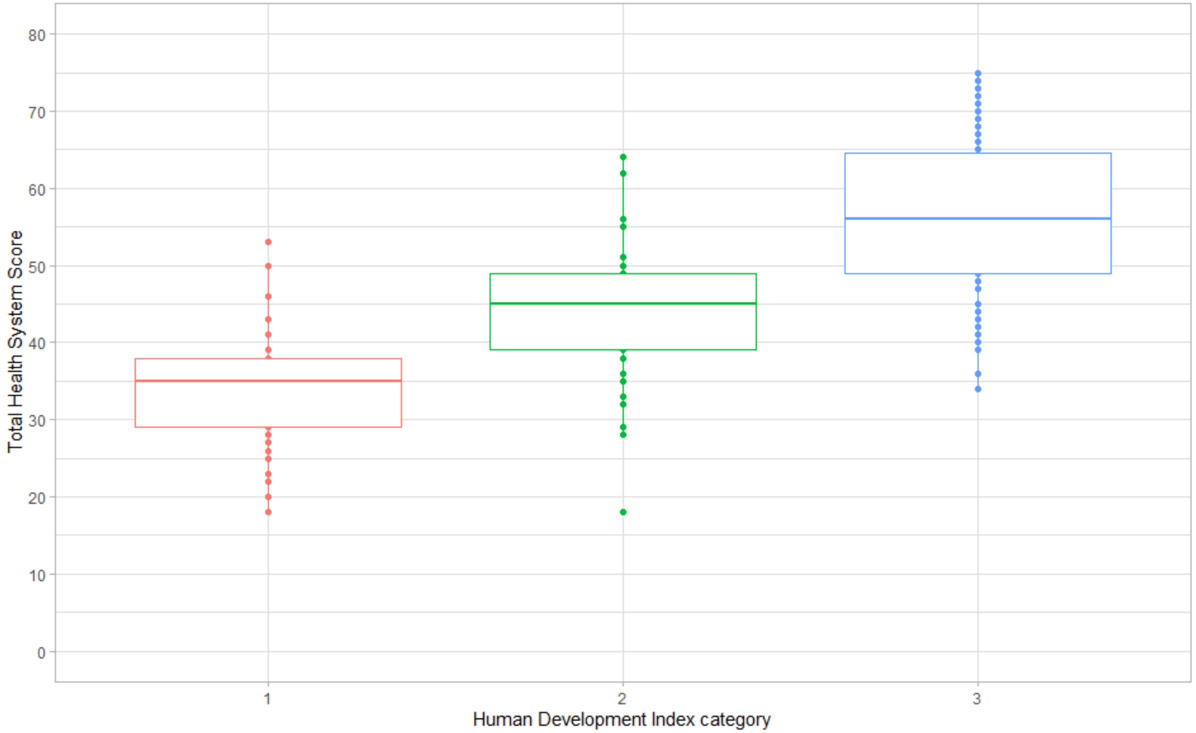

Figure S1: Box plot showing total health system scores across different categories of Human development index.
1= Low HDI index: Countries with HDI score <0.550; 2=Medium HDI index: Countries with HDI score between 0.550 and 0.699; 3=High HDI index: Countries with HDI score above 0.800

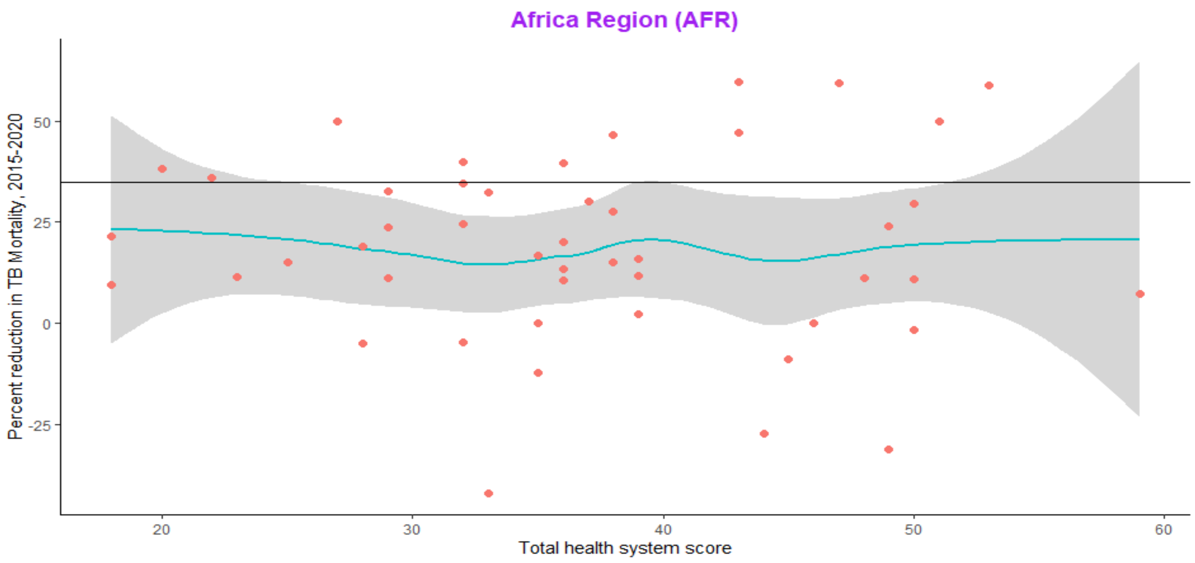

Africa Region (AFR)
Percent reduction in TB Mortality, 2015-2020
50
25
0
-25
20
30
40
50
60
Total health system score

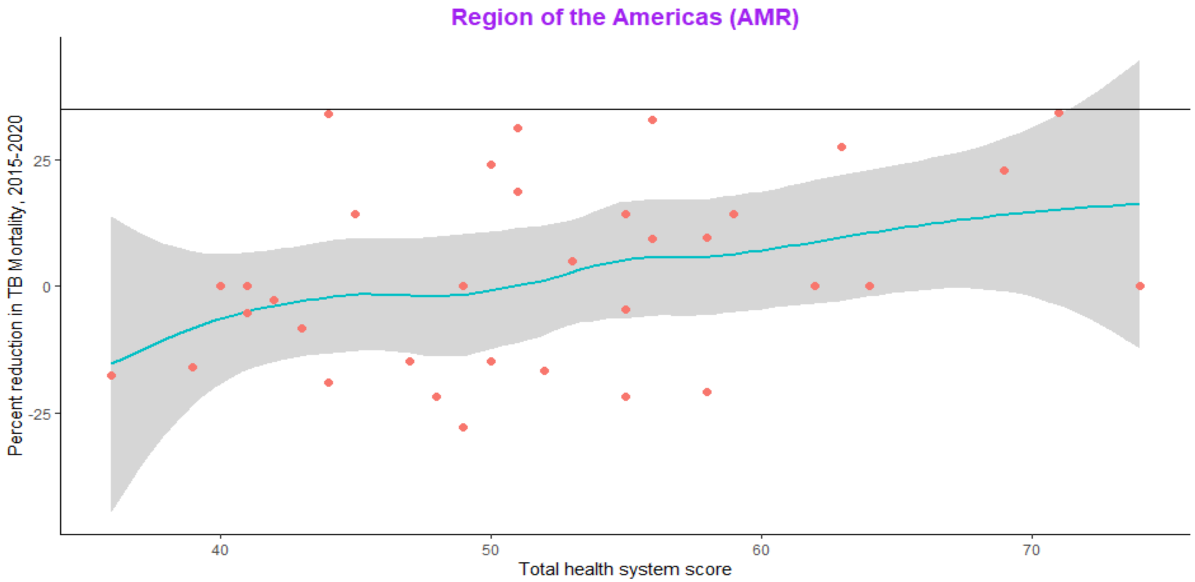

Region of the Americas (AMR)
Percent reduction in TB Mortality, 2015-2020
25
0
-25
40
50
60
70
Total health system score

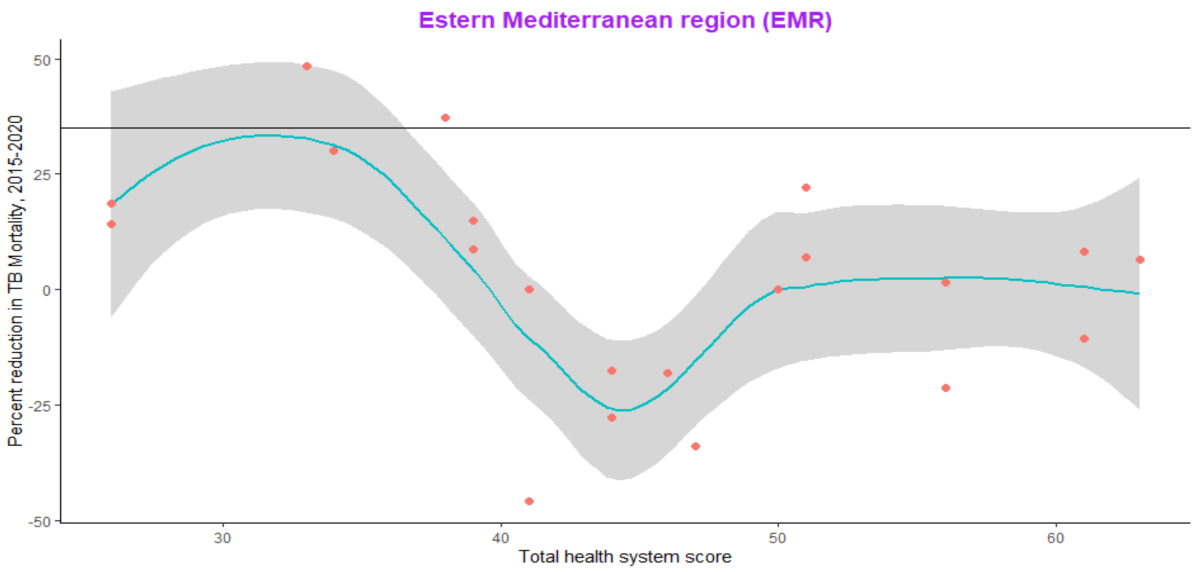
Estern Mediterranean region (EMR)
Percent reduction in TB Mortality, 2015-2020
50
25
0
-25
-50
30
40
50
60
Total health system score

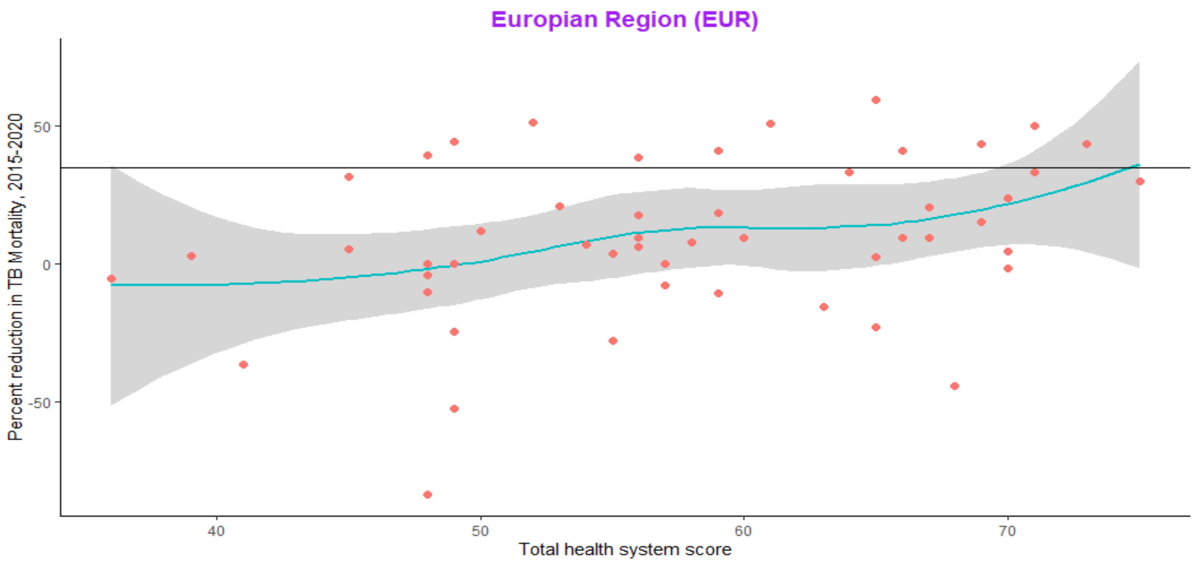
Europian Region (EUR)
Percent reduction in TB Mortality, 2015-2020
50
0
-50
40
50
60
70
Total health system score

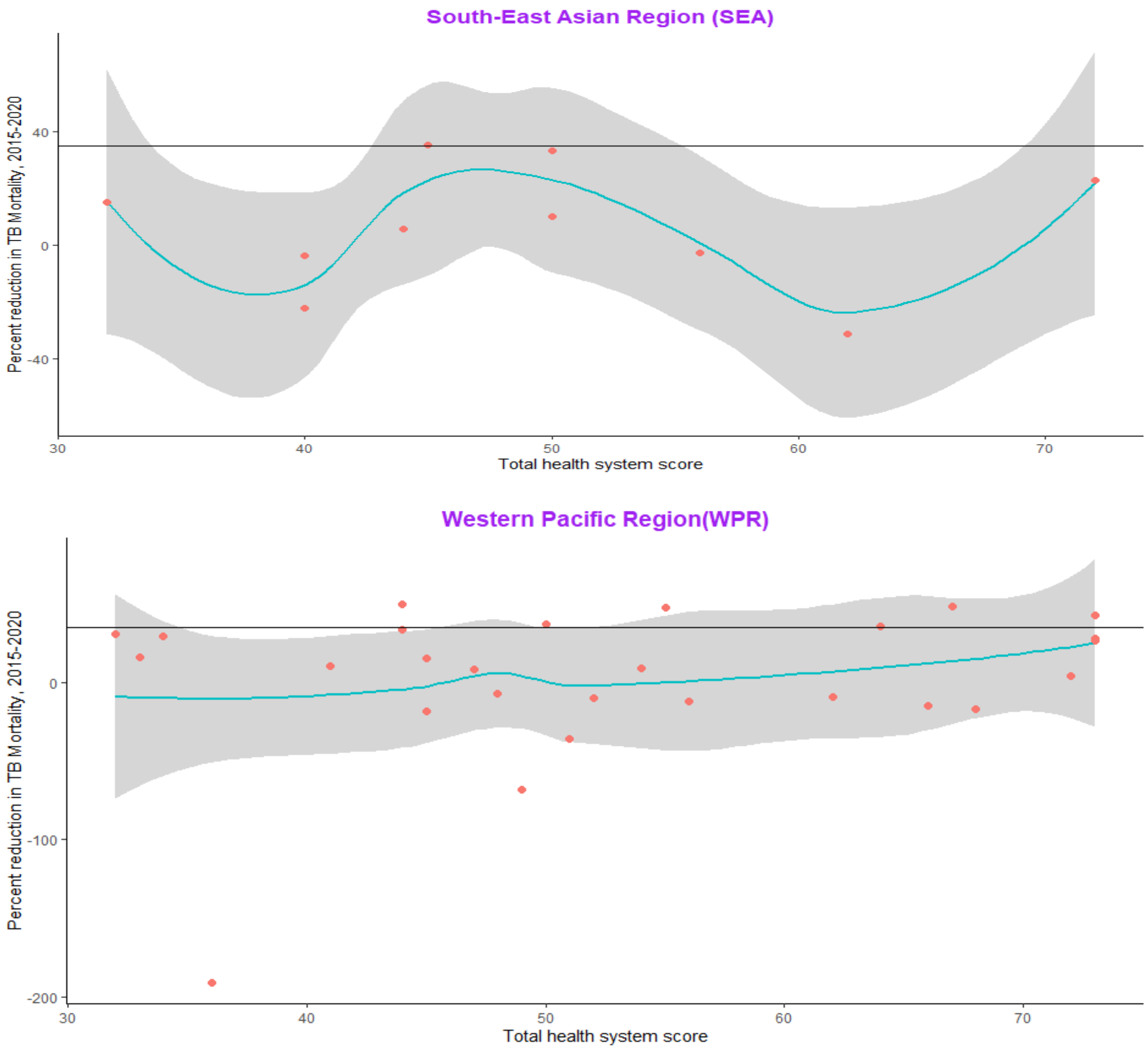

Figure S2: Kernel-weighted local polynomial smoothed relationship between percent reduction in TB mortality and total health systems scores, for each WHO region
